# Discovery of the Anticancer Activity for Lung and Gastric Cancer of a Brominated Coelenteramine Analog

**DOI:** 10.3390/ijms23158271

**Published:** 2022-07-27

**Authors:** Patricia González-Berdullas, Renato B. Pereira, Cláudia Teixeira, José Pedro Silva, Carla M. Magalhães, José E. Rodríguez-Borges, David M. Pereira, Joaquim C. G. Esteves da Silva, Luís Pinto da Silva

**Affiliations:** 1 Centro de Investigação em Química (CIQUP), Instituto de Ciências Moleculares (IMS), Departamento de Geociências, Ambiente e Ordenamento do Território, Faculdade de Ciências, Universidade do Porto, Rua do Campo Alegre s/n, 4169-007 Porto, Portugal; patricia.berdullas@fc.up.pt (P.G.-B.); up201705721@edu.fc.up.pt (J.P.S.); up201201533@edu.fc.up.pt (C.M.M.); jcsilva@fc.up.pt (J.C.G.E.d.S.); 2 REQUIMTE/LAQV, Laboratory of Pharmacognosy, Department of Chemistry, Faculty of Pharmacy, University of Porto, R. Jorge Viterbo Ferreira, 228, 4050-313 Porto, Portugal; rjpereira@ff.up.pt (R.B.P.); pg40748@alunos.uminho.pt (C.T.); 3 LAQV/REQUIMTE, Departamento de Química e Bioquímica, Faculdade de Ciências, Universidade do Porto, Rua do Campo Alegre s/n, 4169-007 Porto, Portugal; jrborges@fc.up.pt; 4 LACOMEPHI, GreenUPorto, Departamento de Geociências, Ambiente e Ordenamento do Território, Faculdade de Ciências, Universidade do Porto, Rua do Campo Alegre s/n, 4169-007 Porto, Portugal

**Keywords:** cancer, Coelenterazine, Coelenteramine, Coelenteramide, bioluminescence, chemiluminescence, anticancer therapy

## Abstract

Cancer is still a challenging disease to treat, both in terms of harmful side effects and therapeutic efficiency of the available treatments. Herein, to develop new therapeutic molecules, we have investigated the anticancer activity of halogenated derivatives of different components of the bioluminescent system of marine Coelenterazine: Coelenterazine (**Clz**) itself, Coelenteramide (**Clmd**), and Coelenteramine (**Clm**). We have found that **Clz** derivatives possess variable anticancer activity toward gastric and lung cancer. Interestingly, we also found that both brominated **Clmd** (**Br-Clmd**) and **Clm** (**Br-Clm**) were the most potent anticancer compounds toward these cell lines, with this being the first report of the anticancer potential of these types of molecules. Interestingly, **Br-Clm** possessed some safety profile towards noncancer cells. Further evaluation revealed that the latter compound induced cell death via apoptosis, with evidence for crosstalk between intrinsic and extrinsic pathways. Finally, a thorough exploration of the chemical space of the studied **Br-Clm** helped identify the structural features responsible for its observed anticancer activity. In conclusion, a new type of compounds with anticancer activity toward gastric and lung cancer was reported and characterized, which showed interesting properties to be considered as a starting point for future optimizations towards obtaining suitable chemotherapeutic agents.

## 1. Introduction

Cancer is one of the most problematic diseases of our time, having been responsible for the estimated death of 9.6 million patients in 2018 [1]. While some developments [2] led to improvements in the survival rates for different cancer types, many patients still cannot escape therapy failure [2]. Furthermore, more common approaches (such as chemotherapy) can cause serious side effects [1], as they are not able to sufficiently distinguish between tumor and nontumor cells. However, even more selective approaches are not without relevant side effects (e.g., skin problems or autoimmune reactions) [3,4,5]. Additionally, some targeted therapies are not useful for all patients while adding other problems such as therapeutic resistance [3,4,5]. Thus, more effective and safe anticancer drugs are needed.

Photodynamic therapy (PDT) has the potential to be an innovative therapy due to its minimally invasive nature, the fast-healing rate of healthy tissues, and its fewer side effects [6,7]. In PDT, a photosensitizer accumulates in tumor tissue, and is activated by light to generate highly cytotoxic singlet oxygen [6,8]. While PDT is selective, the low penetration depth of light into biologic tissues restricts its use to the treatment of superficial tumors or tumors in the outer lining of internal organs and cavities [9,10]. Given this, the development of alternative strategies for eliminating the dependency of PDT on external light sources is a hot topic [9,11].

**Figure 1:**
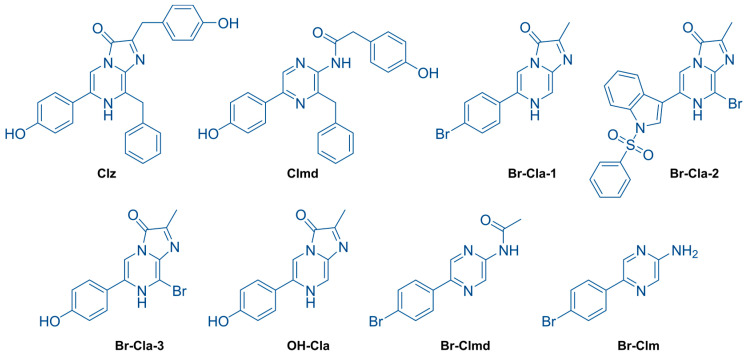
Structures of naturally occurring Coelenterazine (**Clz**) and Coelenteramide (**Clmd**), and their synthetic derivatives **Br-Cla-1/2/3**, **OH-Cla**, **Br-Clmd**, and **Br-Clm** [12,13,14].

Some members of this team have been recently focused on designing strategies for obtaining an intracellular PDT effect, without external light sources, which are based on the chemiluminescent (CL) system of marine Coelenterazine (**Clz**, Figure 1) [12,13,14]. CL consists in the conversion of thermal energy into excitation energy, leading to light emission [11]. In the case of **Clz** (Figure 1), the CL reaction is caused by oxygenation of its imidazopyrazinone core into a high-energy cyclic peroxide intermediate (dioxetanone), which decomposes rapidly into the chemiluminophore, Coelenteramide (**Clmd**, Figure 1). Relevantly, the CL of **Clz** can be solely induced by superoxide anion, a reactive oxygen species (ROS) usually overexpressed in tumor cells [15]. **Clz** can also be responsible for the bioluminescence of many marine organisms (in combination with luciferase enzymes and photoproteins) [11,16].

The **Clz** system can be potentially used in a light-free PDT process because the decomposition of the dioxetanone intermediate allows for the direct chemiexcitation from the singlet ground state to excited states [16,17,18]. Among them, triplet excited states that can interact with molecular oxygen to generate the highly cytotoxic singlet agent (the main agent in PDT), which can occur by activation by a general cancer marker (superoxide anion).

Given this, members of this team have previously developed different brominated **Clz** analogues (**Br-Cla** compounds, Figure 1) [12,13,14]. These compounds showed relevant anticancer activity toward both prostate and breast cancer cells while having no effect on the cellular viability of noncancer cells at the same concentration range [12,13,14]. However, a recent investigation of the anticancer activity of this type of **Br-Cla** compounds revealed that, contrary to the case of breast and prostate cancer, they only showed moderate activity toward neuroblastoma and quite limited activity toward colon cancer [14]. Thus, it is not clear how large the spectrum of application of these molecules toward different cancer types is. Additionally, as we have only investigated CL-capable **Clz** derivatives, we still have not fully determined whether their anticancer activity is indeed related to a superoxide-anion-induced CL reaction or if it is instead related to the intrinsic properties of the halogenated imidazopyrazinone core (present in all **Br-Cla** compounds).

Therefore, to further clarify the spectrum of application of **Br-Cla** compounds, we have investigated for the first time the anticancer activity of previously reported molecules (**Br-Cla-1**, **Br-Cla-2**, and **Br-Cla-3**, Figure 1) [12,13,14] toward both gastric and lung cancer cells. To evaluate the role of the CL reaction itself, we employed two different target-oriented approaches. To evaluate the role of the addition of bromine heteroatoms while maintaining the ability for undergoing a CL reaction, we have also investigated the anticancer activity of a **Br-Cla-1** derivative, **OH-Cla** (Figure 1). Here, the bromine heteroatom is replaced by a hydroxyl group, while the CL-capable imidazopyrazinone core is maintained [18,19]. To assess the role of the CL reaction itself, we developed two brominated and CL-incapable **Br-Cla-1** derivatives. One is its **Clmd** version, **Br-Clmd** (Figure 1), in which the imidazopyrazinone core is substituted by an amidopyrazine one. Finally, we have also developed the Coelenteramine (**Clm**) version of **Br-Cla-1**, **Br-Clm** (Figure 1). **Clms** are both the metabolic products of the bioluminescent reactions of a marine organism employing **Clz** as a substrate [20] and intermediates in the chemical synthesis of **Clz**-based molecules [12,13,14]. As **Br-Clm** and **Br-Clmd** possess an aminopyrazine/amidopyrazine core instead of an imidazopyrazinone one (Figure 1), they are incapable of CL.

The reported results showed that the studied **Br-Cla** compounds possessed relevant anticancer activity toward both gastric and lung cancer cells, pointing to a wide spectrum of application. More importantly, we report the serendipitous and unexpected discovery that **Br-Clm** was the most potent compound toward these cancer cells. Its time- and concentration-dependent profiles were investigated, as well as the involved process of cell death. Finally, its chemical space was thoroughly analyzed. Thus, a new type of molecule with anticancer properties is reported for the first time. It could be considered as a relevant starting point for future optimizations toward obtaining suitable chemotherapeutic agents.

## 2. Results

### 2.1. Br-Cla-1/2/3, *Br-Clm*, and *Br-Clmd* Display Toxicity towards Human Cancer Cells

Our initial set of molecules comprised **Br-Cla-1**/**2**/**3**, **OH-Cla**, **Br-Clm**, and **Br-Clmd**, which were screened for their cytotoxicity towards human cancer cells. Two human cancer cell lines were used, namely gastric (AGS) and non-small cell lung cancer (A549) cells, and the clinically used anticancer drug topotecan was used as a positive control for cytotoxicity. The experiments were initially conducted with 100 µM to identify the most potent molecules.

**Figure 2:**
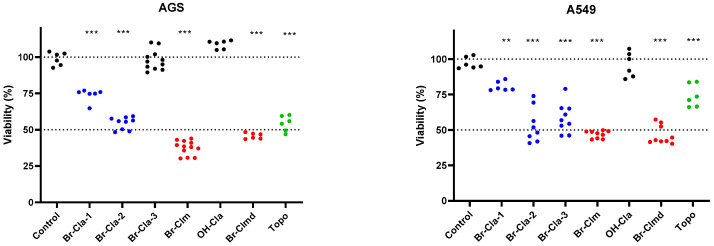
Impact of compounds upon the viability of AGS (left) and A549 (right) cells at 24 h. All molecules were tested at 100 µM, and viability was assessed using the MTT method. Each dot represents an individual determination. Positive control: topotecan (10 µM, Topo). Statistical analysis was conducted between each treatment and the control. ** *p* < 0.01, *** *p* < 0.001.

As shown in Figure 2, **Br-Cla-3** was completely devoid of toxicity in gastric cells (AGS). Substitution of the bromine in the pyrazine ring with a hydroxyl moiety (**OH-Cla**), had no impact in cell viability. Further changes to **OH-Cla**, namely substitution of the phenolic hydroxyl by bromine, resulted in **Br-Cla-1**, with a significant increase in toxicity, reducing cell viability by 25%. Inclusion of a phenylsulfonyl-substituted indole in the structure yielded **Br-Cla-2**, which reduced cell viability by 50%. **Br-Clm**, bearing an amine in the pyrazine ring, induced the most pronounced reduction in cell viability of over 50%. Relevantly, changing the amine group in **Br-Clm** for an amide (**Br-Clmd**) had no appreciable effect on toxicity. The results were similar for lung cancer cells (A549) except in the case of **Br-Cla-3**, which was active and reduced cell viability by around 40%. However, **Br-Clm** was still the most active molecule.

Considering these results, we decided to continue our studies using only **Br-Clm** and **Br-Clmd**, as they were the most potent compounds and presented similar activity in terms of toxicity towards cancer cells.

### 2.2. Cytotoxicity of *Br-Clm* and *Br-Clmd* Is Time- and Concentration-Dependent

**Figure 3:**
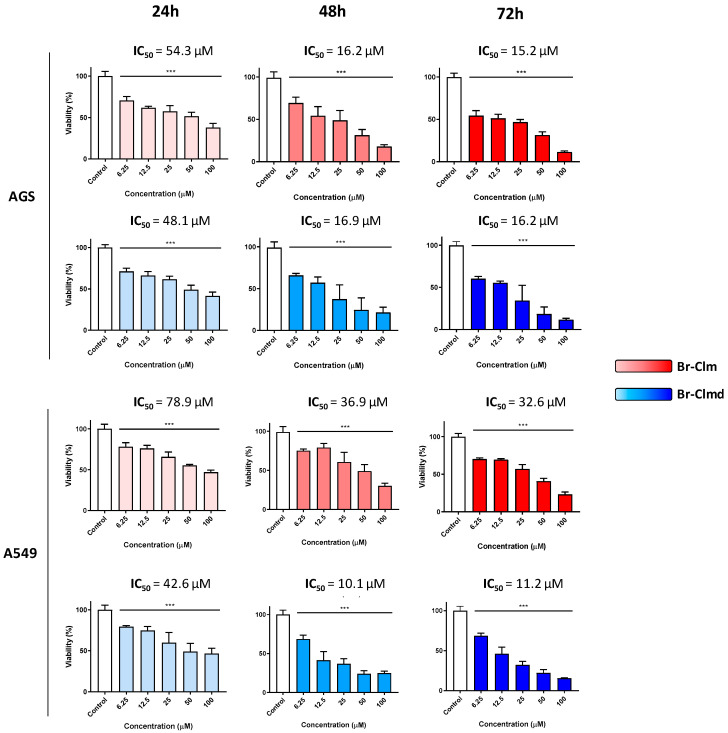
Viability of AGS and A549 cells exposed to **Br-Clm** (red) or **Br-Clmd** (blue) in the 6.25–100 µM range for 24, 48, and 72 h. Statistical analysis was conducted between each treatment and the control. *** *p* < 0.001.

Considering the promising results found earlier, we decided to study the effect of **Br-Clm** and **Br-Clmd** through different concentrations and time-points (Figure 3). When tested in the AGS cell line, **Br-Clm** had an IC_50_ of 54.3 µM and **Br-Clmd** of 48.1 µM, both displaying a concentration-dependent effect in the 6.25–100 µM range. In the case of **Br-Clm**, a marked decrease of the IC_50_ was found as the incubation period increased, with an IC_50_ of 16.2 μM at 48 h which did not change significantly after 72 h (15.2 µM). A nearly equal reduction in the IC_50_ was found for **Br-Clmd**, which had an IC**_50_** of 16.9 μM at 48 h and 16.2 μM at 72 h. The results also showed that the maximum effect was reached after 48 h and that both molecules displayed similar potency. In the case of non-small lung cancer cells, **Br-Clm** was less potent than towards gastric cells (Figure 3), while **Br-Clmd** was slightly more active than what had been found for AGS cells.

**Figure 4:**
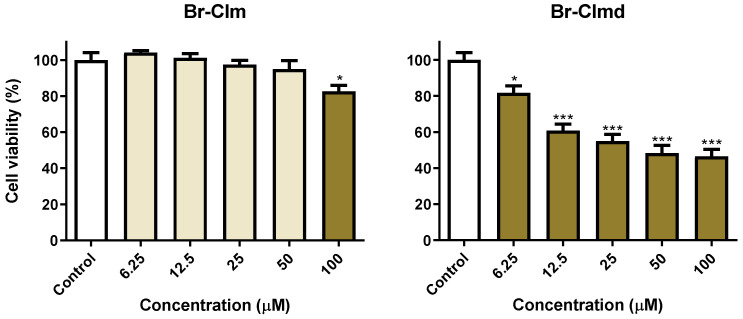
Viability of HaCaT cells exposed to **Br-Clm** or **Br-Clmd** in the 6.25–100 μM range for 24 h. Statistical analysis was conducted between each treatment and the control. * *p* < 0.05, *** *p* < 0.001.

After some preliminary data on the effect of **Br-Clm** and **Br-Clmd** towards gastric and lung cancer cells was compiled, we were interested in assessing the selectivity of the compounds. To this end, we repeated the cytotoxicity studies in the same concentration range, now using human noncancer cells, specifically keratinocytes (HaCaT cells). As shown in Figure 4, although both compounds displayed similar effects towards cancer cells, marked differences were found towards noncancer ones. While **Br-Clm** only displayed residual toxicity (around 10% at the highest tested concentration), **Br-Clmd** was shown to be rather toxic towards noncancer cells, causing nearly 50% viability loss at 25–100 µM. For this reason, we abandoned **Br-Clmd** and continued our studies solely with **Br-Clm**.

### 2.3. *Br-Clm* Triggers Cancer Cell Death via Apoptosis

Considering the marked effect **Br-Clm** triggered in cancer cell viability, we were interested in elucidating the process of cell death that was taking place. To this end, we first evaluated its impact in the morphology of AGS cancer cells. Cells were exposed to the compound for 24 h, after which they were stained with phalloidin and DAPI to stain actin and chromatin, respectively.

**Figure 5:**
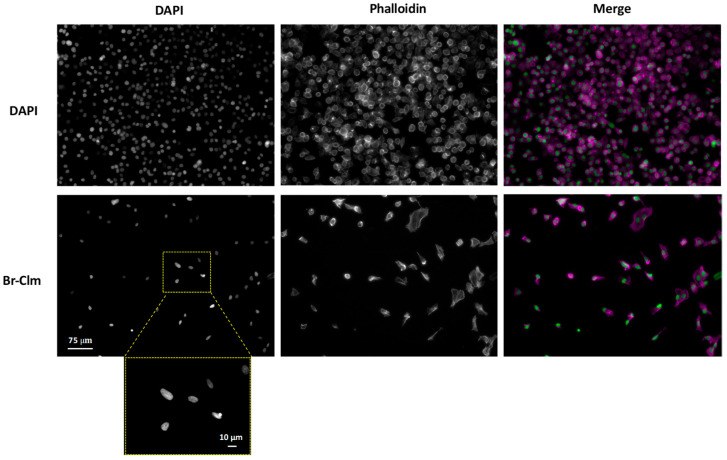
Morphological assessment of the effect of **Br-Clm** (100 µM) upon AGS cells after 24 h of incubation. Cell morphology was evaluated using phalloidin (cytoplasmic traits) and DAPI (chromatin status). Objective: 20×/40× (insert).

As can be seen in Figure 5, incubation of the cells with **Br-Clm** resulted in a marked reduction in cell density, in agreement with the results from cell viability. As seen in the DAPI panel, cells treated with **Br-Clm** displayed smaller and odd-shaped nuclei, which could suggest that a process of organized cell death may have been taking place according to published guidelines [21] and our own previous reports [22,23,24].

**Figure 6:**
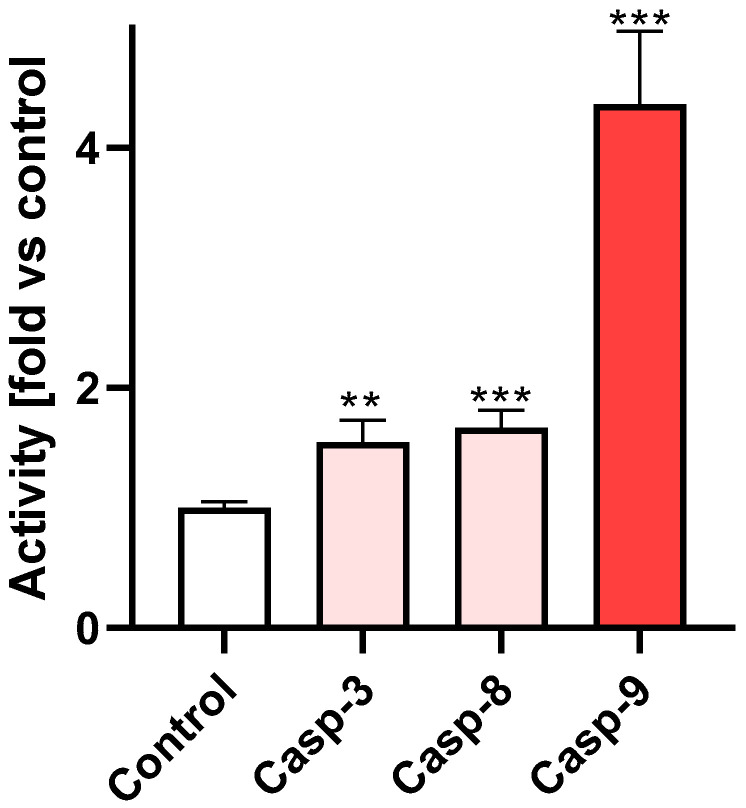
Activity of caspase-3/8/9 in AGS cells after incubation with **Br-Clm** (100 μM) for 24 h when compared to basal levels. ** *p* < 0.01, *** *p* < 0.001.

Among these, apoptosis was the most frequent. Given the involvement of several caspase isoforms in apoptosis, we evaluated the activation status of the effector caspase-3 in cells incubated with **Br-Clm**. As shown in Figure 6, the compound caused a statistically significant increase in the activity of caspase-3, -8, and -9, which points to a crosstalk between the intrinsic and extrinsic pathways of apoptosis.

### 2.4. Exploring the *Br-Clm* Chemical Space

**Figure 7:**
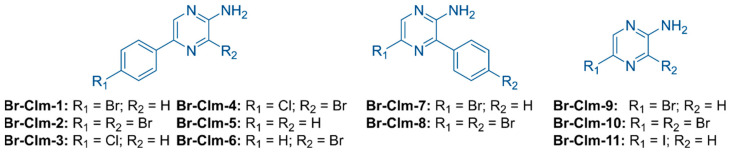
Structures of the screened compounds.

Considering the promising activity and selectivity of **Br-Clm**, we developed an additional set of molecules towards understanding the impact of the chemical structure in the biological activity recorded. We synthesized seven analogs either derived from or inspired by **Br-Clm** (**Br-Clm-2** to **Br-Clm-8**) and an iodine-substituted pyrazin-2-amine fragment (**Br-Clm-11**) and included two commercially available modified pyrazin-2-amine analogs (**Br-Clm-9** and **Br-Clm-10**) (Figure 7). These analogs can be thought as belonging to three different groups (Figure 7), according to their structure and the reasoning for their development. Analogs **Br-Clm-2** to **Br-Clm-6** (Figure 7) possessed the same general structure of **Br-Clm-1** and were developed to evaluate the need for a halogen heteroatom in R_1_ (**Br-Clm-5**), if the bromine heteroatom at R_1_ could be replaced by another halogen (**Br-Clm-3** and **Br-Clm-4**) and/or the effect of increasing the number of halogen heteroatoms (**Br-Clm-2**, **Br-Clm-4**, and **Br-Clm-6**). Analogs **Br-Clm-7** and **Br-Clm-8** (Figure 7) were developed to evaluate the importance of the position of the phenyl moiety for the anticancer activity of these compounds. Analogs **Br-Clm-9** to **Br-Clm-11** (Figure 7) were developed to better assess if the phenyl moiety is indeed needed for the anticancer activity of these compounds, or if direct halogenation of the aminopyrazine core is enough.

These compounds were then evaluated in AGS and A549 cells at the same concentration to compare their cytotoxic effects.

**Figure 8:**
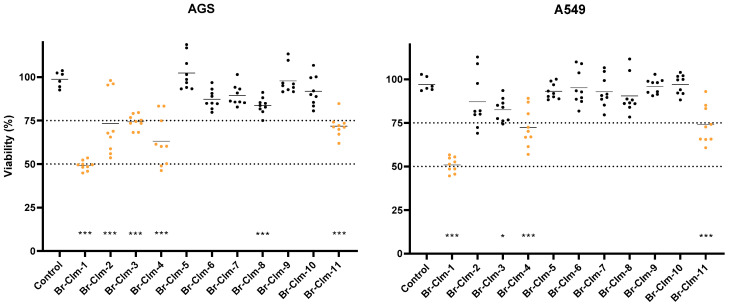
Effect of **Br-Clm** (renamed **Br-Clm-1**) analogs upon the viability of AGS (left) and A549 (right) cells at 24 h. All compounds were tested at 100 µM, and viability was assessed using the MTT method. Each dot represents an individual determination. Statistical analysis was conducted between each treatment and the control. * *p* < 0.05, *** *p* < 0.001.

In both cell lines, substitution of the bromine in **Br-Clm** for chlorine (**Br-Clm-3**) resulted in a significant toxicity decrease, thus pointing to the importance of that halogen in the toxicity profile of these molecules (Figure 8). Introduction of an additional bromine atom in the aminopyrazine ring (**Br-Clm-2**) resulted in a similar effect, even when the compound possessed a chlorine heteroatom in the phenyl ring (**Br-Clm-4**). Albeit to a different extent among both cell lines, with AGS cells being more susceptible to the toxicity of the compounds. Importantly, the only halogen-free analog (**Br-Clm-5**) was completely devoid of toxicity in both cell lines. This importance of the halogen is shown to be site-specific, as the inclusion of a bromine in the pyrazine ring still resulted in an inactive molecule (**Br-Clm-6**). Simplified derivatives of **Br-Clm**, namely single-ring molecules such as **Br-Clm-9** and **Br-Clm-10**, were also inactive, however, the iodine-bearing **Br-Clm-11** displayed mild toxicity (Figure 8).

**Figure 9:**
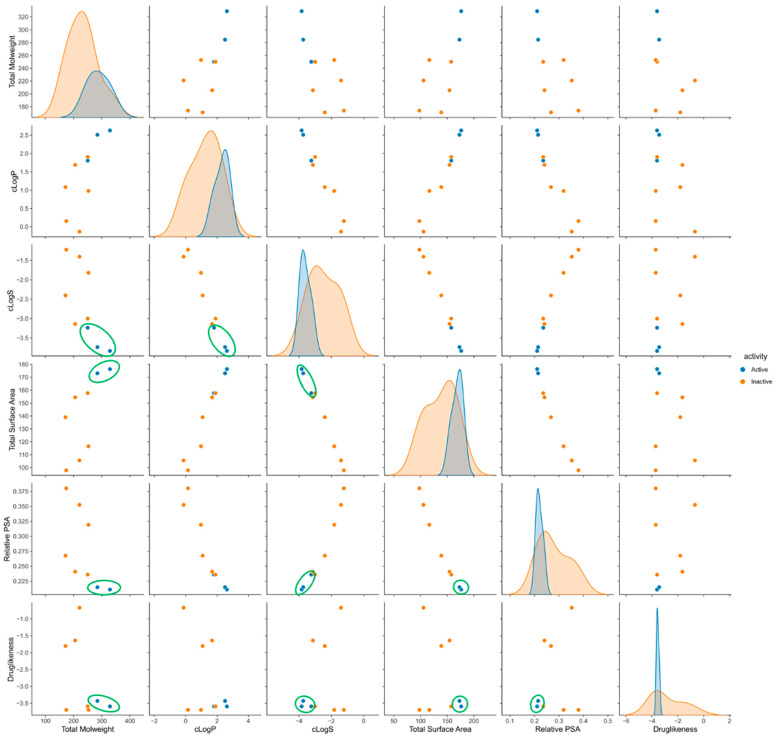
Pair plot representation of selected chemical features of **Br-Clm** derivatives. Orange: inactive towards AGS cells; blue: top three active molecules.

To further understand the chemical space occupied by **Br-Clm** derivatives, we computed several descriptors and physicochemical properties and classified them as “active” or “inactive” based on their impact on the viability of AGS cells and as *per* the results of Figure 8. As shown in Figure 9, the ten **Br-Clm** derivatives occupied a wide chemical space with different properties. We chose the three most active molecules and, as shown, several combinations of properties showed that they clustered together. These results suggest that the combined study of chemometrics and experimentally determined biological activity can guide the future development of these drugs by selecting their ideal chemical characteristics to direct their subsequent synthesis and bioactivity.

It is known that potential new drugs tend to possess lower attrition rates during clinical trials if they conform to Lipinski’s rule of five. The **Br-Clm** compounds are already in line in terms of hydrogen bond donors/acceptors and molecular mass (lower than 500 Da). The remaining criterion is having a logP not greater than 5, with the optimum range being between 0 and 3 [25]. Analysis performed in Figure 9 showed that these compounds presented a logP lower than 5, meaning that the compounds followed the criteria of Lipinski’s rule of five.

Another parameter of interest for the successful development of potential new drugs is their longer-term stability. This parameter was measured for the **Br-Clm** compounds in methanolic solutions by monitoring intensity variations by both absorbance (Figures S21 and S22) and fluorescence (Figures S23 and S24) measurements. Initial measurements were performed in Week 1, followed by weekly measurements (Weeks 2, 3, and 4), with samples being stored at 4 °C between analyses.

By absorbance measurements (Figure S22), all compounds appeared to be quite stable over the analysis period, with minor absorbance intensity variations in the latter weeks mainly for **Br-Clm-9/10/11**. However, when longer-term stability was evaluated by fluorescence (Figure S24), significant variation was now found for several compounds. Namely, there was a large fluorescence enhancement over the weeks for **Br-Clm-2/4/6/9/10/11**, and a relevant decrease in light emission for **Br-Clm-7**. Meanwhile, the fluorescence signal for **Br-Clm-1/3/5/8** was quite stable, in agreement with absorbance measurements. These results indicated that, for starters, fluorescence measurements were more sensitive to the stability/instability of the compounds. Furthermore, while **Br-Clm-1/3/5/8** were quite stable during this analysis period, **Br-Clm-2/4/6/7/9/10/11** were decomposing somewhat quickly, with changes already seen between Week 1 and Week 2 (Figure S24). Nevertheless, given that absorbance measurements did not find relevant weekly variations (Figure S22), this instability should not result from significant structural changes (which should be detected by UV-Vis spectroscopy).

We have also tried to evaluate the shorter-term stability of the compounds in buffer solutions at pH 5.2 and 7.4, which tried to mimic a tumor environment (associated with an acidic pH) and nontumor cells (associated with a neutral pH). Given that longer-term stability assays indicated that fluorescence measurements were more sensitive than absorbance ones, shorter-term stability was only assessed using the former technique (Figures S25–S28). Initial measurements were performed on Day 1, followed by daily measurements on Days 2 and 3. Between measurements, the samples were left at room temperature.

At pH 5.2 (Figure S26), most of the compounds presented quite stable fluorescence signals with the exception of **Br-Clm-4/6/8**. For **Br-Clm-4/6**, there was a fluorescence enhancement, while for **Br-Clm-8,** there was a reduction of the light emission intensity. Thus, most of the compounds presented relevant stability in the buffer solution (pH 5.2), indicating that they should be stable in a tumor environment (a good indicative of their anticancer potential). Most of the compounds **(**Figure S28) were also quite stable at neutral pH, with the exception of **Br-Clm-2/4/6/8**. Once again, the instability was also typically correlated with fluorescence enhancement over time, with exception of **Br-Clm-8**. Thus, these results indicated that most of the **Br-Clm** compounds should be stable in biological media, which can help to explain the time-dependent anticancer activity of **Br-Clm-1**.

It is relevant to highlight that instability was typically found for compounds with halogens directly bound to the aminopyrazine core. Furthermore, instability was also generally associated with fluorescence enhancement, and relevant changes were not detected by absorbance measurements. Given this, the results point to this instability being the result of removal, over time, of the halogen directly bound to the aminopyrazine core. This change could be small enough to not be detected by absorbance measurements. Moreover, the introduction of halogens typically decreases fluorescence by enhancing intersystem crossing (ISC) to triplet states through the heavy-atom effect. Thus, removal of the halogen could lead to fluorescence enhancement by eliminating the heavy-atom effect.

## 3. Discussion

Investigation of the cytotoxicity of **Br-Cla** compounds toward tumor cells revealed that they also possessed anticancer activity toward both gastric and lung cancer (Figure 2). Particularly, **Br-Cla-2** induced ~50% toxicity toward both cell lines, while **Br-Cla-1** induced about 25% toxicity for both gastric and lung cancer. Meanwhile, **Br-Cla-3** was inactive toward gastric cancer, while inducing ~50% toxicity toward lung cancer, demonstrating relevant tumor specificity. These results show that these compounds do possess a relevant spectrum of application, after previously presenting relevant anticancer activity toward neuroblastoma, breast, and prostate cancer [12,13,14].

Interestingly, while **Br-Cla** compounds possess the same imidazopyrazinone core and are brominated (Figure 1), they maintain their anticancer activity with varying substitution degrees. Thus, they are capable of cytotoxic activity with interesting structural flexibility. Nevertheless, the nonbrominated derivative of **Br-Cla-1** (**OH-Cla**, Figure 1), employed as a nonhalogenated control, was inactive toward both gastric and lung cancer (Figure 2). This indicated that the imidazopyrazinone core was not enough to induce cytotoxicity, while highlighting the biological importance of the halogen [14]. Nevertheless, comparison between **Br-Cla-1/2/3** indicated that the halogen did not require to be introduced at a specific position to lead to cytotoxicity. This halogen dependency, without being site-specific, does support our hypothesis that the anticancer activity of **Br-Cla** compounds is related to the CL-induced formation of triplet states, aided by the heavy-atom effect resulting from bromination [12,13,14].

However, cytotoxicity assays (Figure 2) led to the serendipitous finding that both **Br-Clm-1** and **Br-Clmd** (Figure 1) also possessed anticancer activity, more potent even than that presented by **Br-Cla** compounds. As we tested these compounds as brominated non-CL-capable controls, to evaluate the true role of the CL reaction in the anticancer activity, these results could indicate that the activity of **Br-Cla** is not associated with the CL reaction. However, some care must be taken before reaching that conclusion. For one, **Br-Clm-1** and **Br-Clmd** are closer in structural similarity (aminopyrazine and amidopyrazine, Figure 1) than their counterparts, the **Br-Cla** compounds (imidazopyrazinone core, Figure 1). Despite that, there are relevant differences between the cytotoxic potential of **Br-Clm-1** and **Br-Clmd**, which may point to different mechanisms of action. Thus, given these differences, it is doubtful that **Br-Clm-1** and **Br-Clmd** can indeed help explain the anticancer activity of **Br-Cla** compounds, despite the initial consideration regarding their structural similarities.

Given this, this study allowed the serendipitous finding of **Br-Clm-1** as a potential novel chemotherapeutic agent with relevant anticancer activity toward both gastric and lung cancer (Figure 2), reaching IC_50_ values of 15.2 and 32.6 µM (Figure 3), respectively. More relevantly, this compound presented some safety toward noncancer human cells (Figure 4). Furthermore, **Br-Clm-1** was shown to follow Lipinski’s “rule of five” [26], since it has fewer than 5 H-bond donors and 10 H-bond acceptors, its molecular weight did not exceed 500 Da, and its computed logP was below 5. Partition coefficient values ranging from 0 to 3 are desirable in the drug development process, since an excessive lipophilicity (higher logP) can result in lower permeability rates, and a high hydrophilicity (lower logP) is often translated into metabolic or bioavailability issues [25]. Even though there are notable exceptions to Lipinski’s rule for drug likeness [27], it is still a good starting point for drug candidates that are small molecules. This compound was also shown to be stable over both longer storage periods and in the shorter-term in buffer solutions at different pH. Thus, while the actual potency of **Br-Clm-1** might not already be at the desired level, this compound shows enough interesting characteristics that justify its potential as a starting point for future optimizations of this system (in terms of potency) towards obtaining suitable chemotherapeutic agents.

The chemical space of **Br-Clm-1** was thoroughly explored by the synthesis and characterization of several halogenated and nonhalogenated derivatives (Figure 7). Evaluation of their cytotoxicity (Figure 8) revealed that **Br-Clm-1** remained the compound with higher anticancer activity. More importantly, this analysis showed that the *para*-substitution of the aminopyrazine core with a bromophenyl moiety was essential for the activity of these compounds, and that substitution of the bromine by chlorine or *orto*-substitution of the aminopyrazine core negatively affected their anticancer activity (Figure 1 and Figure 8). Elimination of the phenyl moiety and/or changing the phenyl moiety to near the amino group essentially inactivated these compounds (Figure 1 and Figure 8). This indicates that the anticancer mechanism of action of **Br-Clm-1** was heavily dependent on the inclusion of the bromophenyl moiety at a specific position.

It should be noted that, as described in the beginning of this section, the anticancer activity of **Br-Cla** compounds did show a relevant degree of structural flexibility, contrary to **Br-Clm-1** (which requires a bromophenyl moiety at the *para* position). Thus, this is another indicator that the mechanism of action for these two types of compounds may be different, and so, **Br-Clm-1** is not expected to explain the anticancer activity of **Br-Cla** compounds. Thus, future studies should be performed to understand the anticancer activity of both **Br-Cla** and **Br-Clm** compounds and how they effectively differ from each other.

## 4. Materials and Methods

Dimethyl sulfoxide (DMSO), trypan blue, 3-(4,5-dimethylthiazolyl-2)-2,5-diphenyltetrazolium bromide (MTT), and 4′,6-diamidino-2-phenylindole (DAPI) were acquired from Sigma-Aldrich (St. Louis, MO, USA). Phalloidin was purchased from VWR. Dulbecco’s Modified Eagle’s Medium (DMEM), Hank’s balanced salt solution (HBSS), fetal bovine serum (FBS), a penicillin–streptomycin solution (penicillin 10,000 units/mL and streptomycin 10,000 μg/mL), and 0.25% trypsin-EDTA were obtained from GIBCO, Invitrogen™ (Grand Island, NY, USA). A Caspase 3, Caspase 8, and Caspase 9 Multiplex Activity Assay Kit (Fluorometric) was purchased from Abcam (Cambridge, UK). Solvents were purchased from Merck (Darmstadt, Germany) and were either of synthesis- or HPLC-grade.

### 4.1. Synthesis

The synthesis of the compounds started with the functionalization of commercial bromopyrazin-2-amine either with a bromine or iodine atom. A subsequent reaction of the resulting *para*-substituted aminopyrazines **Br-Clm-9** or **Br-Clm-11** with *N*-bromosuccinimide in ethanol provided the homo-(**Br-Clm-10**) or the hetero-dihalogenated products, respectively. Coelenteramines **Br-Clm-1/3/5/7/8** were obtained after the Suzuki–Miyaura cross-coupling of the corresponding functionalized aminopyrazines with commercial boronic acids using bis(triphenylphosphine)palladium (II) dichloride as the palladium source and potassium carbonate as the base. Compounds **Br-Clm-2**, **Br-Clm-4**, and **Br-Clm-6** were obtained after halogenation of Coelenteramines **Br-Clm-1**, **Br-Clm-3**, and **Br-Clm-5** in standard conditions, respectively. Synthetic Coelenteramide **Br-Clmd** was synthesized through *N*-acetylation of **Br-Clm** using pyridine as the base to avoid the formation of the disubstituted subproduct. Precursors of the initial Coelenterazine derivatives **Br-Cla-1/2/3**, **OH-Cla**, and **Br-Clm-1** were synthesized from pyrazin-2-amine in a similar manner, following the procedures described in [12,13,14]. Then, the imidazopyrazinone core was formed after condensation of the corresponding Coelenteramines with a 1,2-dicarbonylic fragment (methyl glyoxal) [12,13,14]. This route has been repeatedly used as the main synthetic path for the obtention of Coelenterazine analogs and derivatives since it goes through stable intermediates, provides the desired compounds in high yields, and is compatible with a great variety of functional groups. The structural characterization was performed using ^1^H- and ^13^C-NMR spectroscopy as well as FT-MS spectrometry. **Br-Cla-1/2/3**, **OH-Cla**, and **Br-Clm-1/3** have already been described in the literature [12,13,14], **Br-Clm-9** and **Br-Clm-10** are commercially available, and further details for the compounds **Br-Clmd** and **Br-Clm-1/2/4/5/6/7/8/11** can be found in the Supplementary Materials.

### 4.2. Photophysical Characterization and Stability Studies

Methanolic stock solutions (5 mM) of the compounds were prepared and stored at –20 °C until use. Absorbance spectra were measured at room temperature with a VWR^®^ UV-Vis Spectrophotometer (UV-3100PC) in a quartz cuvette. Fluorescence spectra were also measured at room temperature using a Horiba Jovin Fluoromax 4 spectrofluorimeter (integration time of 0.1 s and slits of 5 nm) with quartz cuvettes. Absorbance spectra and fluorescence spectra measurements were performed at least in triplicate, using 30 µM solutions of each compound, prepared by dilution of the 5 mM stock solutions. While absorbance spectra measurements were performed only in methanol solutions, fluorescence spectra were obtained in methanol, in sodium acetate buffer pH 5.2, and in phosphate buffer pH 7.4 solutions.

Two types of stability measurements were performed: one focused on long-term stability, and the other on stability in buffer solutions (at two pH values). Longer-term stability was assessed by measuring the initial absorbance and fluorescence spectra of the studied compounds (30 μM in methanolic solutions) in Week 1, followed by weekly measurements (in Weeks 2, 3, and 4). Between measurements, the solutions were stored in a fridge at 4 °C. To assess the short-term stability of the compounds in a buffer solution, the 5 mM methanolic stock solutions of each compound were diluted into 30 µM solutions in either a sodium acetate buffer (pH 5.20) or a phosphate buffer (pH 7.4). Shorter-term stability measurements were performed measuring the initial fluorescence spectra for the studied compounds on Day 1, followed by daily measurements on Days 2 and 3. Between measurements, the solutions were left at room temperature.

### 4.3. Cell Culture

A549, AGS, and HaCaT cell lines were purchased from ATCC (Manassas, VA, USA) and maintained in medium DMEM + GlutaMAX^TM^ with 1% penicillin/streptomycin and 10% FBS at 37 °C in a humidified atmosphere of 5% CO**_2_**. The cells were tested for mycoplasma contamination after thawing, and then every 2 weeks during the entirety of the experiments.

### 4.4. Assessment of Viability—MTT Assay

The cells were seeded in 96-well plates; human non-small cell lung cancer cells (A549) at a density of 10,000 cells/well at 24 h, 5000 cells/well at 48 h, and 2500 cells/well at 72 h; human gastric cancer cells (AGS) were seeded at a density of 15,000 cells/well at 24 h, 10,000 cells/well at 48 h, and 5000 cells/well at 72 h; and HaCaT cells were seeded at 15,000 cells/well at 24 h and allowed to attach for 24 h under the conditions described above, as reported before [28]. After an incubation period of 24, 48, or 72 h with the compounds, a 0.5 mg/mL MTT solution was added and incubated for 2 h. The formazan in each well was dissolved in a solution of 3:1 DMSO/isopropanol. Lastly, the absorbance at 560 nm was read in a Thermo Scientific ™ Multiskan ™ GO microplate reader.

### 4.5. Morphological Analysis

The cells were cultured in 96-well plates at a density indicated above and treated with the compounds under study. The cells were incubated for 24 h, after which they were washed with HBSS and fixed with a 10% formalin solution for 20 min at room temperature. Phalloidin–CF543 (5 U/mL) and DAPI (0.25 µg/mL) were added, and the cells were stained for 30 min at room temperature.

Images were acquired in an inverted Eclipse Ts2R-FL (Nikon) equipped with a Retiga R1 camera and treated with Fiji.

### 4.6. Caspase Activity Assay

AGS cells were plated at the same density mentioned above for the MTT assay and exposed to **Br-Clm** for 24 h, as previously determined [28]. Caspase-3, -8, and -9 activity was assessed using an Abcam’s Caspase Multiplex Activity Assay Kit (Fluorometric). After the incubation time, 50 μL of the supernatant were removed from each well, followed by the addition of 50 μL of a caspase substrate, diluted at 1:200 in an assay buffer. The plate was incubated for 45 min in a humidified incubator at 37 °C with 5% CO_2_, protected from light. After that, fluorescence was read in Cytation 3 at three specific wavelengths: caspase-3 (535–620 nm), caspase-8 (490–525 nm), and caspase-9 (370–450 nm). All results were normalized for DNA content to account for potential differences in cell density arising from cell death. The results corresponded to the fold-increase of fluorescence in treated versus untreated cells of each well for which at least three independent experiments performed in duplicate.

### 4.7. Chemometric Analysis

Physicochemical parameters for all molecules were calculated using DataWarrior [29]. The data was exported to a csv file and analyzed using NumPy [30] and pandas [31] in Python 3.9. Graphics for Figure 9 were generated using seaborn 0.11.2 [32].

### 4.8. Statistical Analysis

In the preliminary assessment of data, outliers were identified by the Grubbs’ test. A Shapiro–Wilks normality test was performed to ensure that all data followed a normal distribution. Comparison between the means of controls and each experimental condition was performed using ANOVA (Dunnett’s multiple comparisons test). Data was expressed as the mean ± standard deviation (SD) of three independent experiments, unless otherwise specified, each performed in triplicate. GraphPad Prism 9.0 software (San Diego, CA, USA) was used, and values were considered statistically significant with a *p* < 0.05.

## 5. Conclusions

Here, we investigated the anticancer potential of the system of marine **Clz** by developing several halogenated analogs of **Clz** itself, **Clmd**, and **Clm**. Their anticancer activity was then evaluated toward both gastric and lung cancer. Among them, **Br-Clm** showed the most potent anticancer activity toward both cancer cell lines. Further evaluation revealed that **Br-Clm** induced cell death via apoptosis, that it also followed Lipinski’s rule of five, and that it was stable over different storage times and conditions. An exploration of its chemical space identified the structural features responsible for its anticancer activity. Thus, a new type of molecule with anticancer activity toward gastric and lung cancer was discovered and characterized for the first time.

## Supplementary Materials

The following supporting information can be downloaded at: https://www.mdpi.com/article/10.3390/ijms23158271/s1.
