# Supplementary material for: Discovery of the Anticancer Activity for Lung and Gastric Cancer of a Brominated Coelenteramine Analog
Source: Int J Mol Sci. 2022 Jul 27;23(15):8271. doi: 10.3390/ijms23158271 (PMC9368541; doi:10.3390/ijms23158271)
Supplement: Supplementary file 1 [file ijms-23-08271-s001.zip › ijms-1817697-supplementary.pdf]

Supplementary Information for

Discovery of the Anticancer Activity for Lung and Gastric Cancer of a Brominated Coelenteramine Analog

Patricia González-Berdullas[1±], Renato B. Pereira[2±], Cláudia Teixeira[2], José Pedro Silva[1], Carla M. Magalhães[1], José E. Rodríguez-Borges[3], David M. Pereira[2,*], Joaquim C.G. Esteves da Silva[1,4], Luís Pinto da Silva[1,4,*]

[1] Chemistry Research Unit (CIQUP), Institute of Molecular Sciences (IMS), Faculty of Sciences, University of Porto, Rua do Campo Alegre 687, 4169-007 Porto, Portugal; patricia.berdullas@fc.up.pt (P.G.-B.); up201705721@edu.fc.up.pt (J.P.A.S.); up201201533@edu.fc.up.pt (C.M.M.);

[2] REQUIMTE/LAQV, Laboratory of Pharmacognosy, Department of Chemistry, Faculty of Pharmacy, University of Porto, R. Jorge Viterbo Ferreira, 228, 4050-313 Porto, Portugal; pg40748@alunos.uminho.pt (C.T.); rjpereira@ff.up.pt (R.B.P.);

[3] LAQV/REQUIMTE, Department of Chemistry and Biochemistry, Faculty of Sciences, University of Porto, Rua do Campo Alegre 697, 4169-007 Porto, Portugal; jrborges@fc.up.pt (J.E.R.-B.);

[4] LACOMEPHI, GreenUPorto, Department of Geosciences, Environment and Territorial Planning, Faculty of Sciences, University of Porto, Rua do Campo Alegre 697, 4169-007 Porto, Portugal; jcsilva@fc.up.pt (J.C.G.E.S.);

* Corresponding authors: luis.silva@fc.up.pt (L.P.d.S.) and dpereira@ff.up.pt (D.M.P.).

± These authors contributed equally to this work.

Table of Contents

1. General Synthetic Procedures S4
   1.1. 5-(4-Bromophenyl)pyrazin-2-amine (**Br-Clm-1**) S5
   1.2. *N*-(5-(4-Bromophenyl)pyrazin-2-yl)acetamide (**Br-Clmd**) S5
   1.3. 3-Bromo-5-(4-bromophenyl)pyrazin-2-amine (**Br-Clm-2**) S6
   1.4. 5-(4-Bromophenyl)pyrazin-2-amine (**Br-Clm-3**) S6
   1.5. 3-Bromo-5-(4-chlorophenyl)pyrazin-2-amine (**Br-Clm-4**) S6
   1.6. 5-Phenylpyrazin-2-amine (**Br-Clm-5**) S7
   1.7. 3-Bromo-5-phenylpyrazin-2-amine (**Br-Clm-6**) S8
   1.8. 5-Bromo-3-iodopyrazin-2-amine S8
   1.9. 5-Bromo-3-phenylpyrazin-2-amine (**Br-Clm-7**) S9
   1.10. 5-Bromo-3-(4-bromophenyl)pyrazin-2-amine (**Br-Clm-8**) S9
   1.11. 5-Iodopyrazin-2-amine (**Br-Clm-11**) S10
2. Supporting Figures S11
   2.1. NMR Spectra S11
   - **Figure S1.** ^{1}H-NMR, DEPT, and ^{13}C-NMR spectra for **Br-Clm-1** S11
   - **Figure S2.** ^{1}H-NMR, DEPT, and ^{13}C-NMR spectra for **Br-Clm-2** S12
   - **Figure S3.** ^{1}H-NMR, DEPT, and ^{13}C-NMR spectra for **Br-Clmd** S13
   - **Figure S4.** ^{1}H-NMR, DEPT, and ^{13}C-NMR spectra for **Br-Clm-4** S14
   - **Figure S5.** ^{1}H-NMR, DEPT, and ^{13}C-NMR spectra for **Br-Clm-5** S15
   - **Figure S6.** ^{1}H-NMR, DEPT, and ^{13}C-NMR spectra for **Br-Clm-6** S16
   - **Figure S7.** ^{1}H-NMR, DEPT, and ^{13}C-NMR spectra for 5-bromo-3-iodopyrazin-2-amine S17
   - **Figure S8.** ^{1}H-NMR, DEPT, and ^{13}C-NMR spectra for **Br-Clm-7** S18
   - **Figure S9.** ^{1}H-NMR, DEPT, and ^{13}C-NMR spectra for **Br-Clm-8** S19
   - **Figure S10.** ^{1}H-NMR, DEPT, and ^{13}C-NMR spectra for **Br-Clm-11** S20

   2.2. FT-MS Spectra S21
   - **Figure S11.** FTMS-ESI (+) spectrum for **Br-Clm-1** S21
   - **Figure S12.** FTMS-ESI (+) spectrum for **Br-Clm-2** S21
   - **Figure S13.** FTMS-ESI (+) spectrum for **Br-Clmd** S22
   - **Figure S14.** FTMS-ESI (+) spectrum for **Br-Clm-4** S22
   - **Figure S15.** FTMS-ESI (+) spectrum for **Br-Clm-5** S23

Figure S16. FTMS-ESI (+) spectrum for **Br-Clm-6** S23

Figure S17. FTMS-ESI (+) spectrum for 5-bromo-3-iodopyrazin-2-amine S24

Figure S18. FTMS-ESI (+) spectrum for **Br-Clm-7** S24

Figure S19. FTMS-ESI (+) spectrum for **Br-Clm-8** S25

Figure S20. FTMS-ESI (+) spectrum for **Br-Clm-11** S25

3. Photophysical characterization 26

Figure S21. Absorbance spectra of the **Br-Clm** compounds in 30 μM methanolic solutions S26

Figure S22. Maximum absorbance intensity of 30 μM methanolic solutions of the **Br-Clm** family, measured weekly S27

Figure S23. Emission spectra of the **Br-Clm** compounds in 30 μM methanolic solutions S28

Figure S24. Maximum emission intensity of 30 μM methanolic solutions of the **Br-Clm** family, measured weekly S29

Figure S25. Emission spectra of the **Br-Clm** compounds in 30 μM aqueous solutions buffered to a pH of 5.2 .... S30

Figure S26. Maximum emission intensity of 30 μM aqueous solutions of the **Br-Clm** family buffered to a pH of 5.2, measured every 24 h S31

Figure S27. Emission spectra of the **Br-Clm** compounds in 30 μM aqueous solutions buffered to biological pH (7.4) S32

Figure S28. Maximum emission intensity of 30 μM aqueous solutions of the **Br-Clm** family buffered to a pH of 7.4, measured every 24 h S33

4. Bibliography S34

1. General Synthetic Procedures

Reagents and solvents were purchased from Merck and used without further purification. All reactions involving oxygen or moisture-sensitive compounds were carried out under dry nitrogen atmosphere. Ice-water and silicon baths were used for reactions at low and high temperatures, respectively, with all reaction temperatures referring to the external bath. Organic extracts were dried over anhydrous Na_2SO_4, filtered and concentrated using a rotary evaporator (Büchi® Rotavapor® R-210, Büchi® B-491 Heating Bath 120V, KNF Neuberger D-79112 Vacuum Pump N 035.1.2 AN.18).

Reactions were monitored by thin-layer chromatography (TLC) using aluminum-backed Merck 60 F_{254} silica gel plates and *n*-hexanes-ethyl acetate solvent systems. After visualization under ultraviolet light at 254 nm and 365 nm, the plates were developed by immersion in a solution containing a mixture of *p*-anisaldehyde (2.5%), acetic acid (1%), and sulfuric acid (3.4%) in 95% ethanol followed by heating. Solid compounds were mixed with SiO_2, redissolved in DCM, and concentrated under reduced pressure before purification through column chromatography using silica gel (Aldrich, 230-400 mesh) and EtOAc-hexanes mixtures. Compounds were systematically named following IUPAC recommendations with ChemDraw v20.0.0.41 (Perkin-Elmer, Waltham, MA, USA).

NMR spectra were recorded in $CDCl_3$ and acetone-d_6 solutions on a Bruker NMR spectrometer (Bruker Advance III 400 MHz Ascend, 9.4 Tesla), and chemical shifts are reported on the δ scale (ppm) using the residual solvent signals [δ = 7.26 ppm (^{1}H, $CDCl_3$); δ = 77.0 ppm (^{13}C, t, $CDCl_3$)] and as [δ = 2.05 ppm (^{1}H, qu, acetone-d_6); δ = 29.9 ppm (^{13}C, hep, acetone-d_6), 206.7 ppm (^{13}C, s, acetone-d_6)] internal standards. Coupling constants (*J*) are reported in Hz. FT-MS analysis were done on a LTQ OrbitrapTM XL hybrid mass spectrometer (Thermo Fischer Scientific, Bremen, Germany) controlled by LTQ Tune Plus and Xcalibur 2.1.0.

bs = broad singlet; DCM = Dichloromethane; DMF = Dimethylformamide; DMSO = Dimethyl sulfoxide; ESI = Electrospray ionization; EtOAc = Ethyl acetate; EtOH = Ethanol; FTMS = Fourier transform mass spectrometry; hep = heptet; NBS = *N*-Bromosuccinimide; NIS = *N*-Iodosuccinimide; NMR = Nuclear magnetic resonance; py = Pyridine; qu = quintet; *rt* = Room temperature; s = singlet; t = triplet; THF = Tetrahydrofuran; TLC: Thin layer chromatography.

The synthetic procedures and structural characterization details for **Br-Cla-2** and **Br-Cla-3** can be consulted in reference [1], and for **OH-Cla** in reference [2].

1.1. 5-(4-Bromophenyl)pyrazin-2-amine (Br-Clm-1)

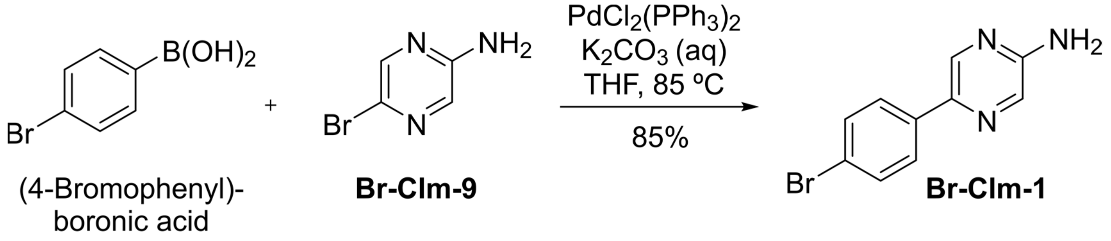

An aqueous solution of K_2CO_3 (1 M, 18.673 mmol, 7.5 equiv) was added to a solution of (4-bromophenyl)boronic acid (0.500 g, 2.490 mmol, 1 equiv) and 5-bromopyrazin-2-amine (**Br-Clm-9**) (0.433 g, 2.490 mmol, 1 equiv) in THF (19 mL) and was deoxygenated with N_2. Then $PdCl_2(PPh_3)_2$ (0.174 g, 0.259 mmol, 0.10 equiv) was added and the resulting mixture was stirred at 85 ºC until no starting material was detected by TLC (1:1 EtOAc-hexanes). The reaction mixture was cooled to room temperature and the aqueous phase discarded. The combined organic layers were washed with brine, dried, and concentrated under reduced pressure. The resulting solid was purified by column chromatography (SiO_2, EtOAc/hexanes gradient) to give 5-(4-bromophenyl)pyrazin-2-amine (**Br-Clm-1**) as pale-yellow solid [0.529 g, 85%, R_f = 0.19 (50% EtOAc/hexanes)].

^{1}H NMR (400 MHz, $CDCl_3$) δ = 8.46 – 8.35 (d, *J*=1.5, 1H), 8.06 – 7.97 (d, *J*=1.5, 1H), 7.76 – 7.71 (m, 2H), 7.57 – 7.51 (m, 2H), 4.93 – 4.45 (bs, 2H). **^{13}C NMR** (101 MHz, $CDCl_3$) δ = 153.5 (C), 141.8 (C), 138.9 (CH), 136.0 (C), 132.0 (2xCH), 131.8 (CH), 127.2 (2xCH), 122.5 (C). **FTMS-ESI (+):** m/z: calcd for $[C_{10}H_9BrN_3]^+$: 249.9980 $[M+H]^+$; found 249.9977 $[C_{10}H_9{}^{79}BrN_3]^+$, 251.9953 $[C_{10}H_9{}^{81}BrN_3]^+$.

1.2. *N*-(5-(4-Bromophenyl)pyrazin-2-yl)acetamide (Br-Clmd)

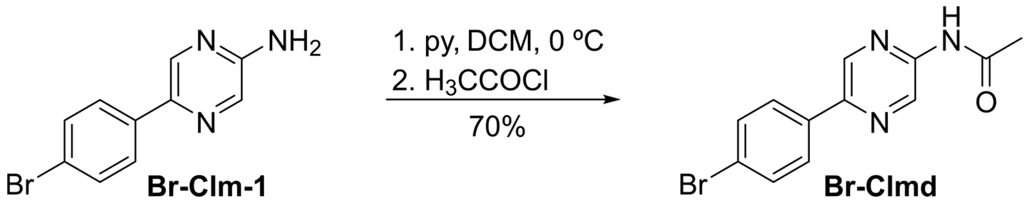

Pyridine (0.087 mL, 1.071 mmol, 1.75 eq) was added to a solution of -(4-bromophenyl)pyrazin-2-amine (**Br-Clm-1**) (0.153 g, 0.612 mmol, 1 eq) in DCM (7.5 mL), which was previously cooled to 0 ºC, and stirred at that temperature for 5 min. Then acetyl chloride (0.130 mL, 1.835 mmol, 3 eq) was added and the mixture stirred at *rt* until no starting material was detected by TLC (1:1 EtOAc-hexanes). The reaction mixture was washed with brine and extracted with DCM. The combined organic layers were dried with anhydrous sodium sulfate, filtered, and concentrated under reduced pressure to give a yellowish solid, which was purified by column chromatography (SiO_2, hexanes-EtOAc gradient) to afford *N*-(5-(4-bromophenyl)pyrazin-2-yl)acetamide (**Br-Clmd**) as a white solid [0.125 g, 70%, R_f = 0.48 (50% EtOAc/hex)].

^{1}H NMR (400 MHz, Acetone) δ = 9.79 (s, 1H), 9.50 (d, *J*=1.5, 1H), 8.87 (d, *J*=1.6, 1H), 8.11 – 7.92 (m, 2H), 7.78 – 7.55 (m, 2H), 2.25 (s, 3H). **^{13}C NMR** (101 MHz, Acetone) δ = 170.0 (C=O), 149.1 (C), 146.9 (C), 140.1 (CH), 136.7 (C), 136.4 (CH), 133.0 (CH),

129.0 (CH), 123.9 (CH), 24.2 (CH_3). **FTMS-ESI (+)**: m/z: calcd for $[C_{12}H_{11}BrN_3O]^+$: 292.0085 $[M+H]^+$; found 292.0090 $[C_{12}H_{11}{}^{79}BrN_3O]^+$, 294.0063 $[C_{12}H_{11}{}^{81}BrN_3O]^+$.

1.3. 3-Bromo-5-(4-bromophenyl)pyrazin-2-amine (Br-Clm-2)

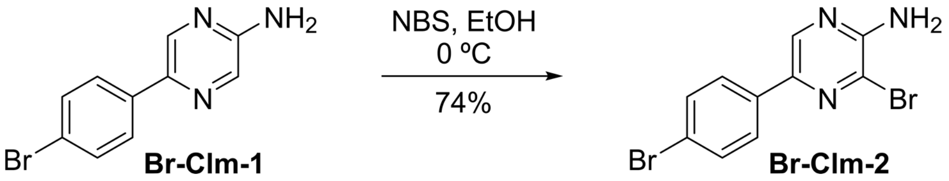

NBS (0.111 g, 0.624 mmol, 1.2 eq) was added to a solution of 5-(4-bromophenyl)pyrazin-2-amine (**Br-Clm-1**) (0.130 g, 0.519 mmol, 1 eq) in ethanol (5 mL), which was previously cooled to 0 ºC, and stirred at that temperature for 15 min. The reaction mixture was then diluted with EtOAc and washed with brine. The combined organic layers were dried with anhydrous sodium sulfate, filtered, and concentrated under reduced pressure to give a brown solid, which was purified by column chromatography (SiO_2, hexanes-EtOAc gradient) to afford 3-bromo-5-(4-bromophenyl)pyrazin-2-amine (**Br-Clm-2**) as a pale-yellow solid [0.127 g, 74 %, R_f = 0.68 (50% EtOAc/hex)].

^{1}H NMR (400 MHz, $CDCl_3$) δ = 8.38 – 8.34 (s, 1H), 7.77 – 7.72 (m, 2H), 7.60 – 7.54 (m, 2H), 5.23 (bs, 2H). **^{13}C NMR** (101 MHz, $CDCl_3$) δ = 151.6 (C), 142.1 (C), 137.6 (CH), 134.6 (C), 132.2 (2xCH), 127.3 (2xCH), 126.0 (C), 123.1 (C). **FTMS-ESI (+)**: m/z: calcd for $[C_{10}H_8Br_2N_3]^+$: 327.9085 $[M+H]^+$; found 327.9078 $[C_{10}H_8{}^{79}Br_2N_3]^+$, 329.9057 $[C_{10}H_8{}^{81}Br_2N_3]^+$, 331.9037 $[C_{10}H_8{}^{79}Br^{81}BrN_3]^+$.

1.4. 5-(4-Bromophenyl)pyrazin-2-amine (Br-Clm-3)

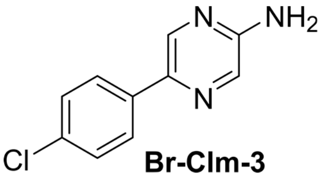

Br-Clm-3 was synthesized following the procedures described in [2]. Structural details are available in said reference.

1.5. 3-Bromo-5-(4-chlorophenyl)pyrazin-2-amine (Br-Clm-4)

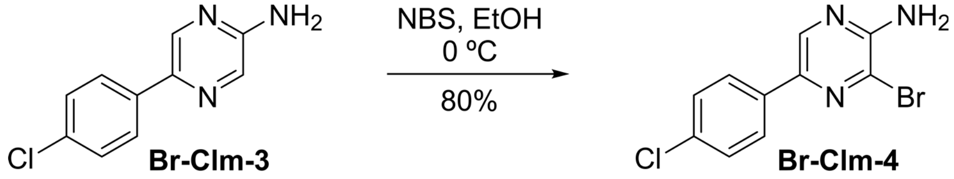

NBS (0.173 g, 0.9725 mmol, 1 eq) was added to a solution of 5-(4-chlorophenyl)pyrazin-2-amine (**Br-Clm-3**) (0.200 g, 0.9725 mmol, 1 eq) in ethanol (10 mL), which was previously cooled to 0 ºC, and stirred at that temperature for 15 min. The reaction mixture

was then diluted with EtOAc and washed with brine. The combined organic layers were dried with anhydrous sodium sulfate, filtered, and concentrated under reduced pressure to give a brown solid, which was purified by column chromatography (SiO_2, hexanes-EtOAc gradient) to afford 3-bromo-5-(4-chlorophenyl)pyrazin-2-amine (**Br-Clm-4**) as a yellowish solid [0.221 g, 80 %, R_f = 0.59 (50% EtOAc/hexanes)].

^{1}H NMR (400 MHz, $CDCl_3$) δ = 8.38 – 8.35 (s, 1H), 7.83 – 7.79 (m, 2H), 7.43 – 7.39 (m, 2H), 5.26 (bs, 2H). **^{13}C NMR** (101 MHz, $CDCl_3$) δ = 151.4 (C), 142.0 (C), 137.1 (CH), 134.8 (C), 134.0 (C), 129.2 (2xCH), 127.0 (2xCH), 126.2 (C). **FTMS-ESI (+)**: m/z: calcd for $[C_{10}H_8BrClN_3]^+$: 283.9590 [M+H]$^+$; found 283.9591 $[C_{10}H_8{}^{79}Br^{35}ClN_3]^+$, 285.9570 $[C_{10}H_8{}^{79}Br^{37}ClN_3]^+$, and $[C_{10}H_8{}^{81}Br^{35}ClN_3]^+$, 287.9510 $[C_{10}H_8{}^{81}Br^{37}ClN_3]^+$.

1.6. 5-Phenylpyrazin-2-amine (Br-Clm-5)

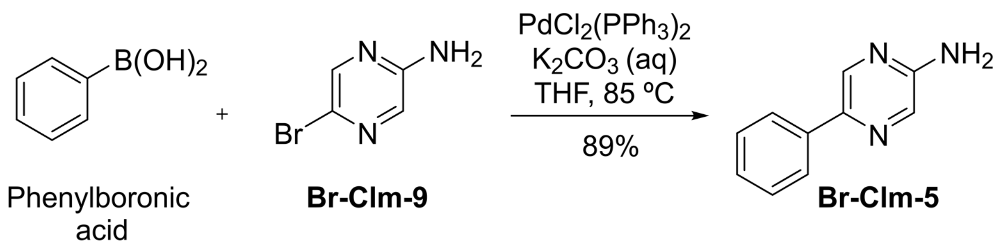

An aqueous solution of K_2CO_3 (1 M, 17.535 mmol, 7.5 equiv) was added to a solution of phenylboronic acid (0.285 g, 2.338 mmol, 1 equiv) and 5-bromopyrazin-2-amine (**Br-Clm-9**) (0.406 g, 2.338 mmol, 1 equiv) in THF (17.5 mL) and was deoxygenated with N_2. Then $PdCl_2(PPh_3)_2$ (0.164 g, 0.234 mmol, 0.10 equiv) was added and the resulting mixture was stirred at 85 ºC until no starting material was detected by TLC (1:1 EtOAc-hexanes). The reaction mixture was cooled to room temperature and the aqueous phase discarded. The combined organic layers were washed with brine, dried, and concentrated under reduced pressure. The resulting solid was purified by column chromatography (SiO_2, EtOAc/hexanes gradient) to give 5-phenylpyrazin-2-amine (**Br-Clm-5**) as pale-yellow solid [0.356 g, 89 %, R_f = 0.63 (50% EtOAc/hexanes)].

^{1}H NMR (400 MHz, $CDCl_3$) δ = 8.49 – 8.42 (d, *J*=1.5 Hz, 1H), 8.12 – 8.05 (d, *J*=1.6, 1H), 7.90 – 7.85 (m, 2H), 7.48 – 7.41 (m, 2H), 7.39 – 7.34 (m, 1H), 4.82 – 4.56 (s, 2H). **^{13}C NMR** (101 MHz, $CDCl_3$) δ = 153.2 (C), 143.2 (C), 139.2 (CH), 137.1 (C), 131.8 (CH), 129.0 (2xCH), 128.3 (CH), 125.8 (2xCH). **FTMS-ESI (+)**: m/z: calcd for $[C_{10}H_{10}N_3]^+$: 172.0875 [M+H]$^+$; found 172.0867 $[C_{10}H_{10}N_3]^+$.

1.7. 3-Bromo-5-phenylpyrazin-2-amine (Br-Clm-6)

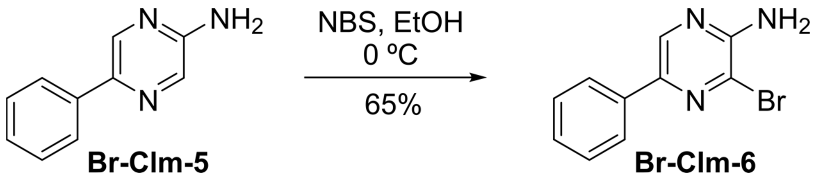

NBS (0.117 g, 0.6546 mmol, 1 eq) was added to a solution of 5-phenylpyrazin-2-amine (**Br-Clm-5**) (0.112 g, 0.6546 mmol, 1 eq) in ethanol (5 mL), which was previously cooled to 0 ºC, and stirred at that temperature for 15 min. The reaction mixture was then diluted with EtOAc and washed with brine. The combined organic layers were dried with anhydrous sodium sulfate, filtered, and concentrated under reduced pressure to give a brown solid, which was purified by column chromatography (SiO_2, hexanes-EtOAc gradient) to afford 3-bromo-5-phenylpyrazin-2-amine (**Br-Clm-6**) as an ochre solid [0.107 g, 65%, R_f = 0.52 (50% EtOAc/hex)].

^{1}H NMR (400 MHz, $CDCl_3$) δ = 8.42 – 8.30 (s, 1H), 7.95 – 7.81 (d, *J*=7.0, 2H), 7.49 – 7.40 (t, *J*=7.4, 2H), 7.41 – 7.31 (m, 1H), 5.17 (bs, 2H). **^{13}C NMR** (101 MHz, $CDCl_3$) δ = 151.3 (CH), 143.4 (C), 137.7 (C), 135.6 (C), 129.0 (CH), 128.8 (CH), 126.0 (C), 125.9 (CH). **FTMS-ESI (+)**: m/z: calcd for $[C_{10}H_9BrN_3]^+$: 249.9980 $[M+H]^+$; found 249.9985 $[C_{10}H_9{}^{79}BrN_3]^+$, 251.9962 $[C_{10}H_9{}^{81}BrN_3]^+$.

1.8. 5-Bromo-3-iodopyrazin-2-amine

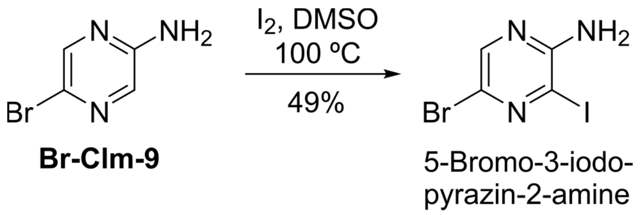

Iodine (0.583 g, 2.298 mmol, 2 eq) was added to a stirring solution of 5-bromopyrazin-2-amine (**Br-Clm-9**) (0.200 g, 1.149 mmol, 1 eq) in DMSO (10 mL) and stirred for 8 h at 100 ºC. The reaction mixture was then cooled to *rt*, poured over an aqueous saturated solution of $Na_2S_2O_3$, and extracted with DCM. The combined organic layers were dried with anhydrous sodium sulfate, filtered, and concentrated under reduced pressure to give a dark brown solid, which was purified by column chromatography (SiO_2, hexanes-EtOAc gradient), affording 5-bromo-3-iodopyrazin-2-amine as a pale-yellow solid [0.169 g, 49 %, R_f = 0.58 (50% EtOAc/hexanes)].

^{1}H NMR (400 MHz, $CDCl_3$) δ = 7.97 (s, 1H), 5.07 (bs, 2H). **^{13}C NMR** (101 MHz, $CDCl_3$) δ = 154.3 (C), 143.3 (CH), 124.5 (C), 101.8 (C). **FTMS-ESI (+)**: m/z: calcd for $[C_4H_4BrIN_3]^+$: 299.8633 $[M+H]^+$; found 299.8632 $[C_4H_4{}^{79}BrIN_3]^+$, 301.8609 $[C_4H_4{}^{81}BrIN_3]^+$.

1.9. 5-Bromo-3-phenylpyrazin-2-amine (Br-Clm-7)

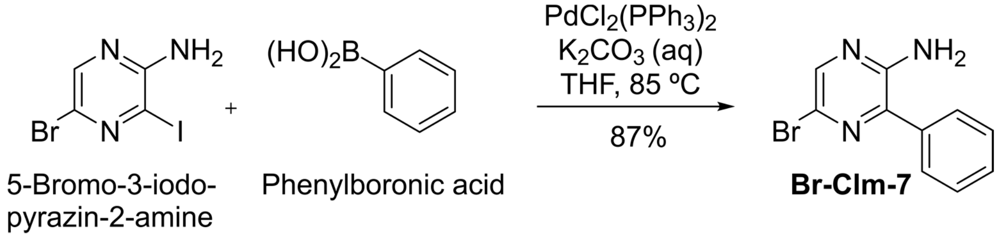

An aqueous solution of K_2CO_3 (1 M, 2.250 mmol, 7.5 equiv) was added to a solution of phenylboronic acid (0.037 g, 0.300 mmol, 1 equiv) and 5-bromo-3-iodopyrazin-2-amine (0.090 g, 0.300 mmol, 1 equiv) in THF (2.5 mL) and was deoxygenated with N_2. Then $PdCl_2(PPh_3)_2$ (0.021 g, 0.030 mmol, 0.10 equiv) was added and the resulting mixture was stirred at 85 ºC until no starting material was detected by TLC (1:1 EtOAc-hexanes). The reaction mixture was cooled to room temperature and the aqueous phase discarded. The combined organic layers were washed with brine, dried, and concentrated under reduced pressure. The resulting solid was purified by column chromatography (SiO_2, hexanes-EtOAc gradient) to give 5-bromo-3-phenylpyrazin-2-amine (**Br-Clm-7**) as an ochre solid [0.065 g, 87 %, R_f = 0.63 (50% EtOAc/hexanes)].

^{1}H NMR (400 MHz, $CDCl_3$) δ = 8.08 – 8.03 (s, 1H), 7.76 – 7.68 (m, 2H), 7.54 – 7.43 (m, 3H), 4.90 (bs, 2H). **^{13}C NMR** (101 MHz, $CDCl_3$) δ = 151.1 (C), 142.1 (CH), 141.3 (C), 135.8 (C), 129.9 (CH), 129.4 (2xCH), 128.2 (2xCH), 127.0 (C). **FTMS-ESI (+)**: m/z: calcd for $[C_{10}H_9BrN_3]^+$: 249.9980 $[M+H]^+$; found 249.9974 $[C_{10}H_9{}^{79}BrN_3]^+$, 251.9953 $[C_{10}H_9{}^{81}BrN_3]^+$.

1.9. 5-Bromo-3-(4-bromophenyl)pyrazin-2-amine (Br-Clm-8)

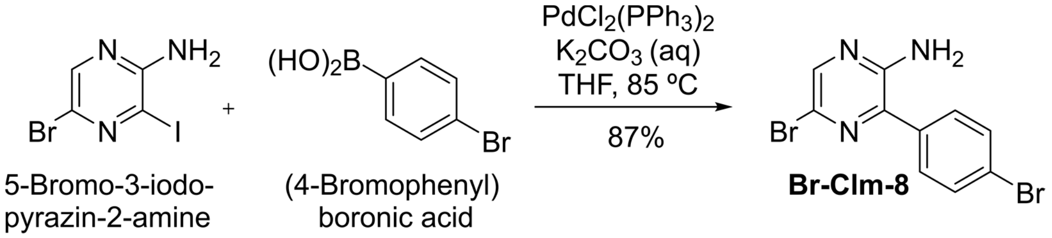

An aqueous solution of K_2CO_3 (1 M, 7.328 mmol, 7.5 equiv) was added to a solution of (4-bromophenyl)boronic acid (0.196 g, 0.977 mmol, 1 equiv) and 5-bromo-3-iodopyrazin-2-amine (0.293 g, 0.977 mmol, 1 equiv) in THF (7.5 mL) and was deoxygenated with N_2. Then $PdCl_2(PPh_3)_2$ (0.069 g, 0.098 mmol, 0.10 equiv) was added and the resulting mixture was stirred at 85 ºC until no starting material was detected by TLC (1:1 EtOAc-hexanes). The reaction mixture was cooled to room temperature and the aqueous phase discarded. The combined organic layers were washed with brine, dried, and concentrated under reduced pressure. The resulting solid was purified by column chromatography (SiO_2, hexanes-EtOAc gradient) to give 5-bromo-3-(4-bromophenyl)pyrazin-2-amine (**Br-Clm-8**) as a yellow solid [0.244 g, 76 %, R_f = 0.76 (50% EtOAc/hexanes)].

^{1}H NMR (400 MHz, $CDCl_3$) δ = 8.08 – 8.07 (s, 1H), 7.68 – 7.58 (m, 4H), 4.94 (bs, 2H). **^{13}C NMR** (101 MHz, $CDCl_3$) δ = 150.9 (C), 142.3 (CH), 140.1 (C), 134.6 (C), 132.6 (2xCH), 129.9 (2xCH), 127.2 (C), 124.3 (C). **FTMS-ESI (+)**: m/z: calcd for $[C_{10}H_8Br_2N_3]^+$: 327.9085 $[M+H]^+$; found 327.9079 $[C_{10}H_8{}^{79}Br_2N_3]^+$, 329.9057 $[C_{10}H_8{}^{79}Br{}^{81}BrN_3]^+$, 331.9037 $[C_{10}H_8{}^{81}Br_2N_3]^+$.

1.10. 5-Iodopyrazin-2-amine (Br-Clm-11)

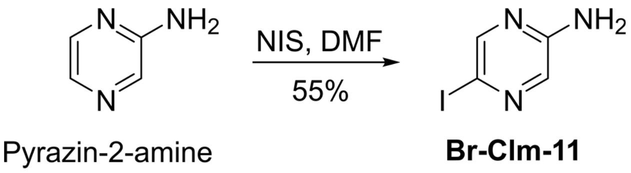

NIS (0.782 g, 3.474 mmol, 1.1 eq) was added to a solution of pyrazin-2-amine (0.300 g, 3.158 mmol, 1 eq) in DMF (20 mL) and stirred at *rt* for 24 h. Then a saturated aqueous solution of $Na_2S_2O_3$ was added, and the mixture was extracted with EtOAc and washed with brine. The combined organic layers were dried with anhydrous sodium sulfate, filtered, and concentrated under reduced pressure to give a brown oil, which was purified by column chromatography (SiO_2, hexanes-EtOAc gradient) to afford 5-iodopyrazin-2-amine (**Br-Clm-11**) as a light yellow solid [0.384 g, 55 %, R_f = 0.48 (50% EtOAc/hex)].

^{1}H NMR (400 MHz, $CDCl_3$) δ = 8.44 – 8.15 (d, *J*=1.5, 1H), 8.05 – 7.70 (d, *J*=1.4, 1H), 4.64 – 4.55 (s, 3H). **^{13}C NMR** (101 MHz, $CDCl_3$) δ = 153.7 (C), 149.7 (CH), 133.6 (CH), 100.6 (C). **FTMS-ESI (+)**: m/z: calcd for $[C_4H_5IN_3]^+$: 221.9528 $[M+H]^+$; found 221.9518.

---

2. Supporting Figures

2.1. NMR Spectra

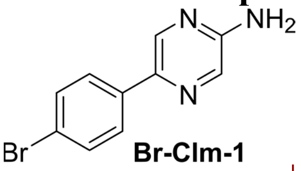

Br-Clm-1

^{1}H NMR (400 MHz, $CDCl_3$) δ = 8.46 – 8.35 (d, *J*=1.5, 1H), 8.06 – 7.97 (d, *J*=1.5, 1H), 7.76 – 7.71 (m, 2H), 7.57 – 7.51 (m, 2H), 4.93 – 4.45 (bs, 2H).

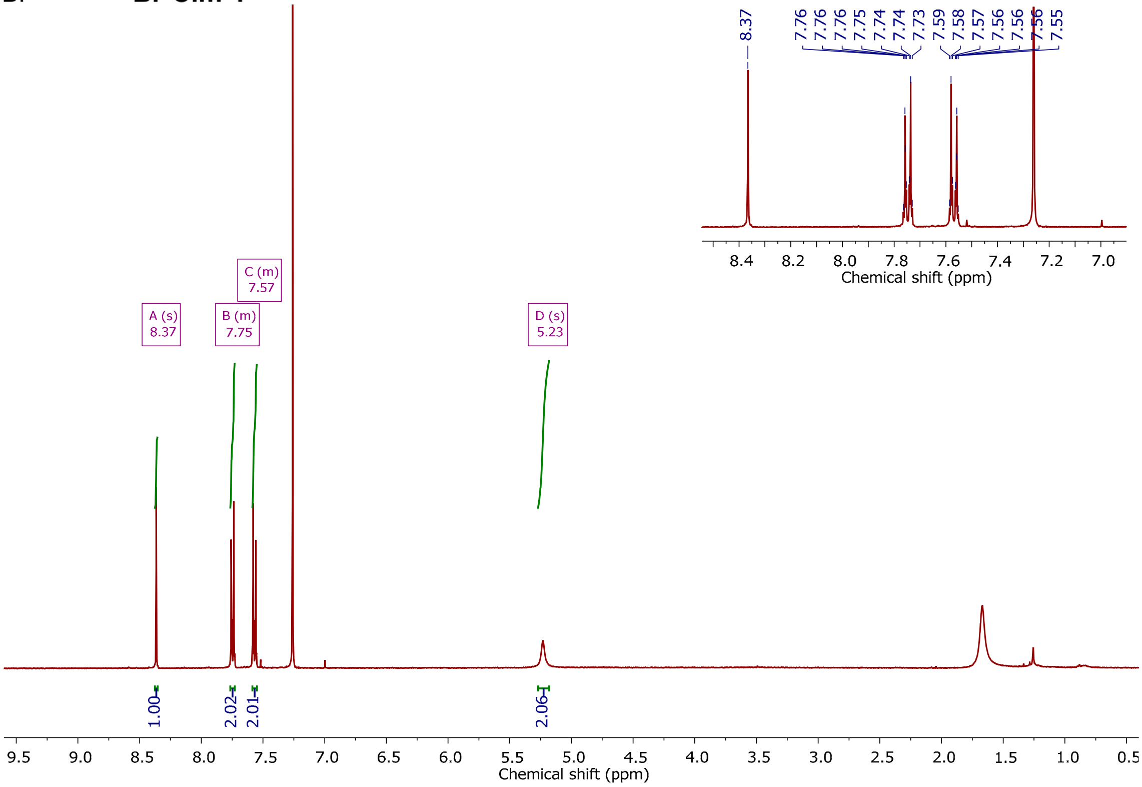

^{13}C NMR (101 MHz, $CDCl_3$) δ = 153.5 (C), 141.8 (C), 138.9 (CH), 136.0 (C), 132.0 (2xCH), 131.8 (CH), 127.2 (2xCH), 122.5 (C).

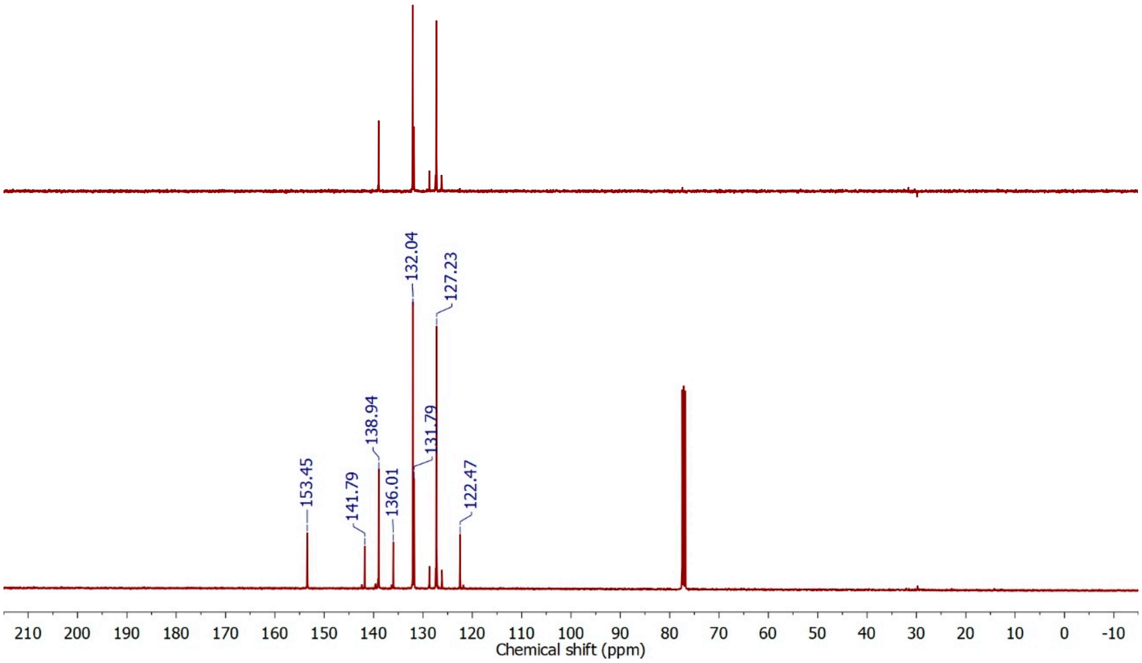

Figure S1. ^{1}H-NMR, DEPT, and ^{13}C-NMR spectra for **Br-Clm-1**.

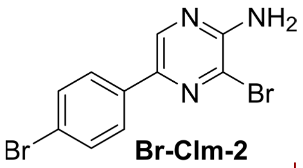

Br-Clm-2

^{1}H NMR (400 MHz, $CDCl_3$) δ = 8.38 – 8.34 (s, 1H), 7.77 – 7.72 (m, 2H), 7.60 – 7.54 (m, 2H), 5.23 (bs, 2H).

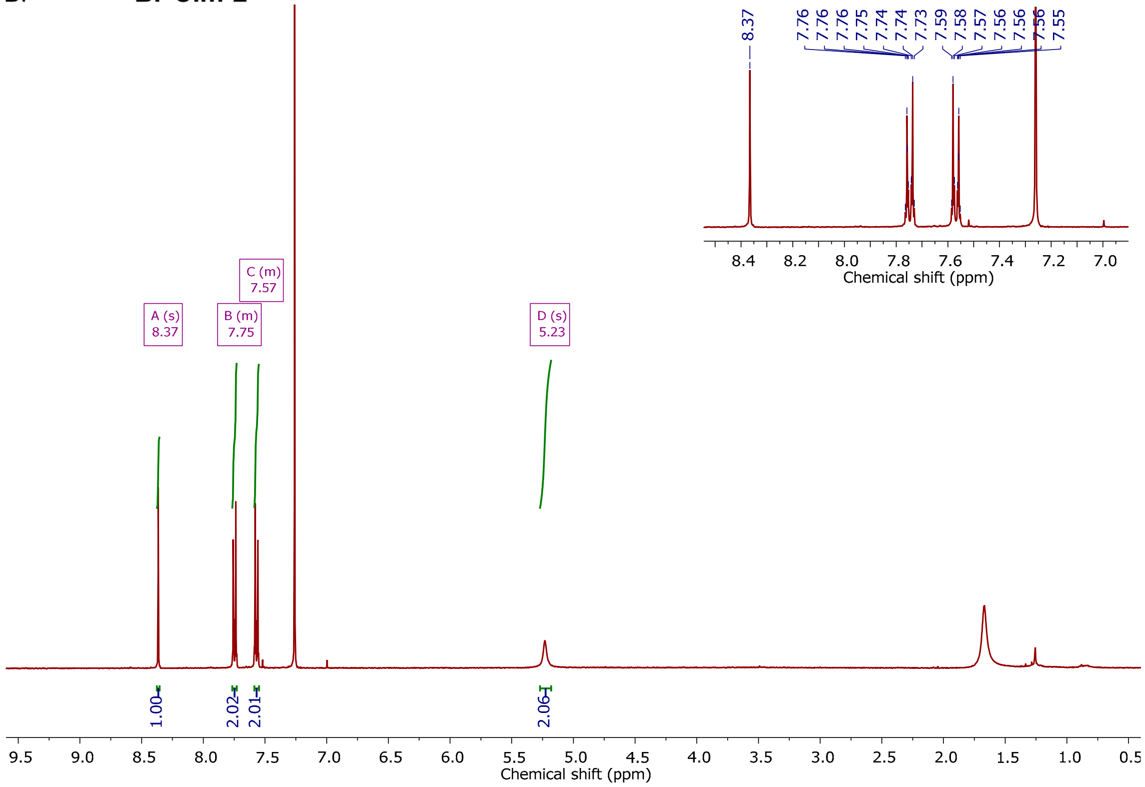

^{13}C NMR (101 MHz, $CDCl_3$) δ = 151.6 (C), 142.1 (C), 137.6 (CH), 134.6 (C), 132.2 (2xCH), 127.3 (2xCH), 126.0 (C), 123.1 (C).

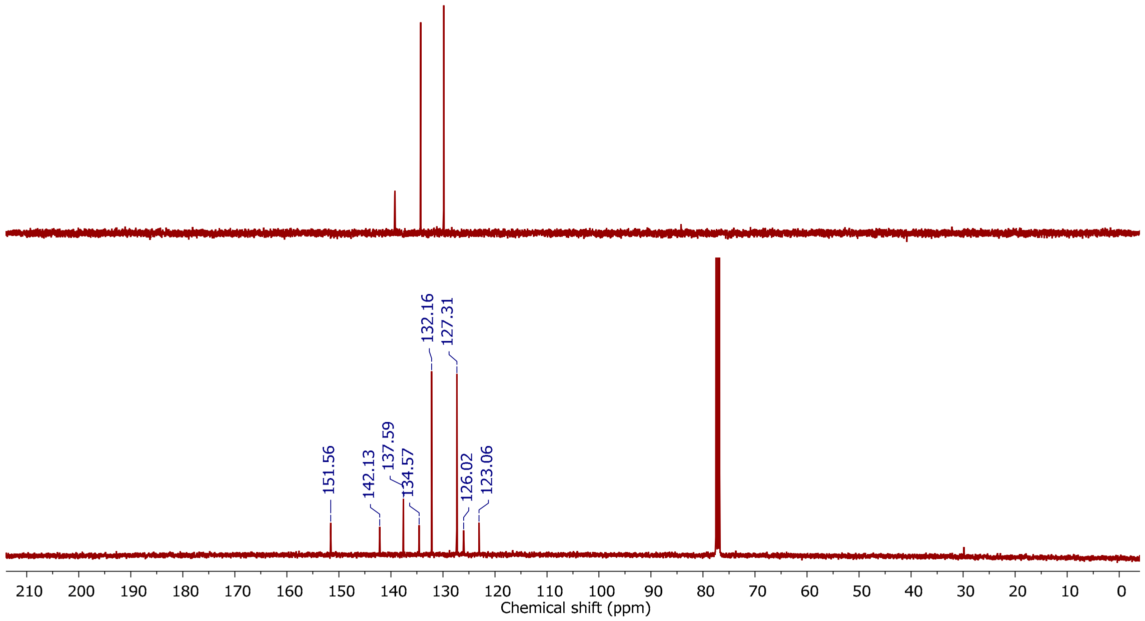

Figure S2. ^{1}H-NMR, DEPT, and ^{13}C-NMR spectra for **Br-Clm-2**.

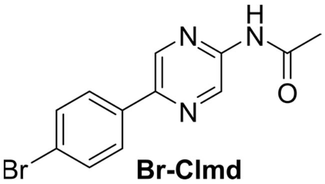

Br-Clmd

[1]**H NMR** (400 MHz, Acetone) δ = 9.79 (s, 1H), 9.50 (d, *J*=1.5, 1H), 8.87 (d, *J*=1.6, 1H), 8.11 – 7.92 (m, 2H), 7.78 – 7.55 (m, 2H), 2.25 (s, 3H).

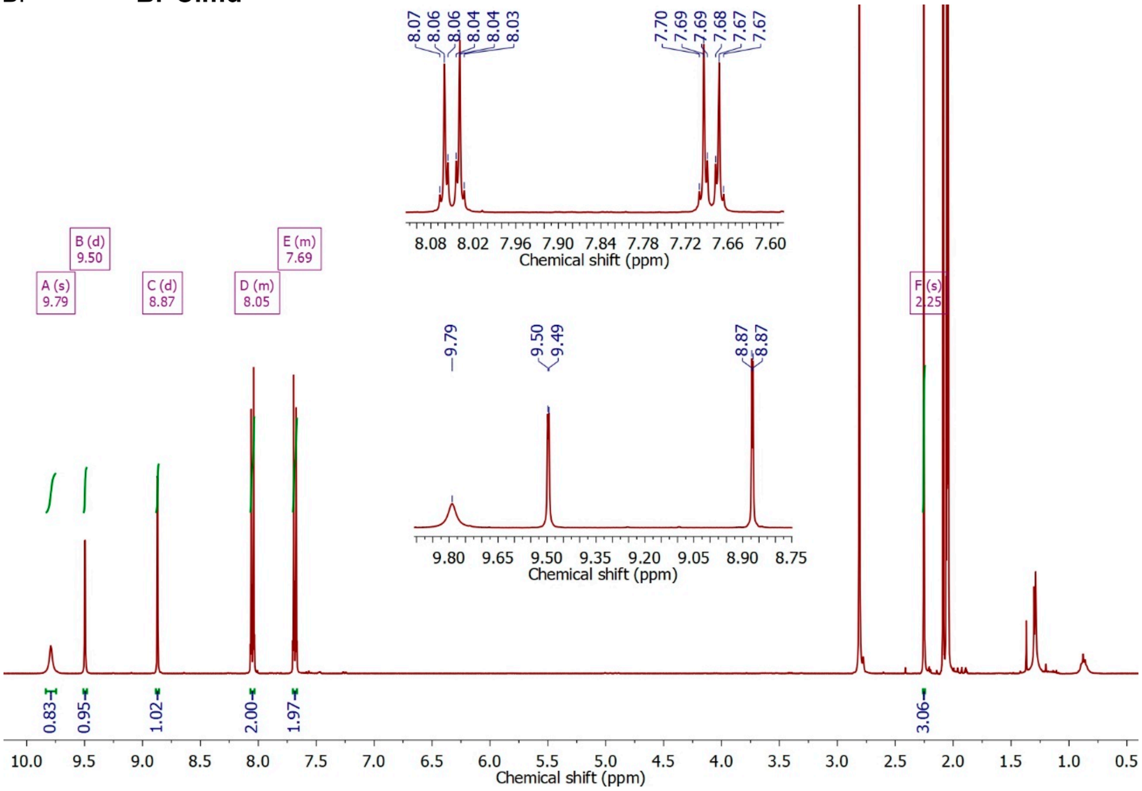

[13]**C NMR** (101 MHz, Acetone) δ = 170.0 (C=O), 149.1 (C), 146.9 (C), 140.1 (CH), 136.7 (C), 136.4 (CH), 133.0 (CH), 129.0 (CH), 123.9 (CH), 24.2 (CH_3).

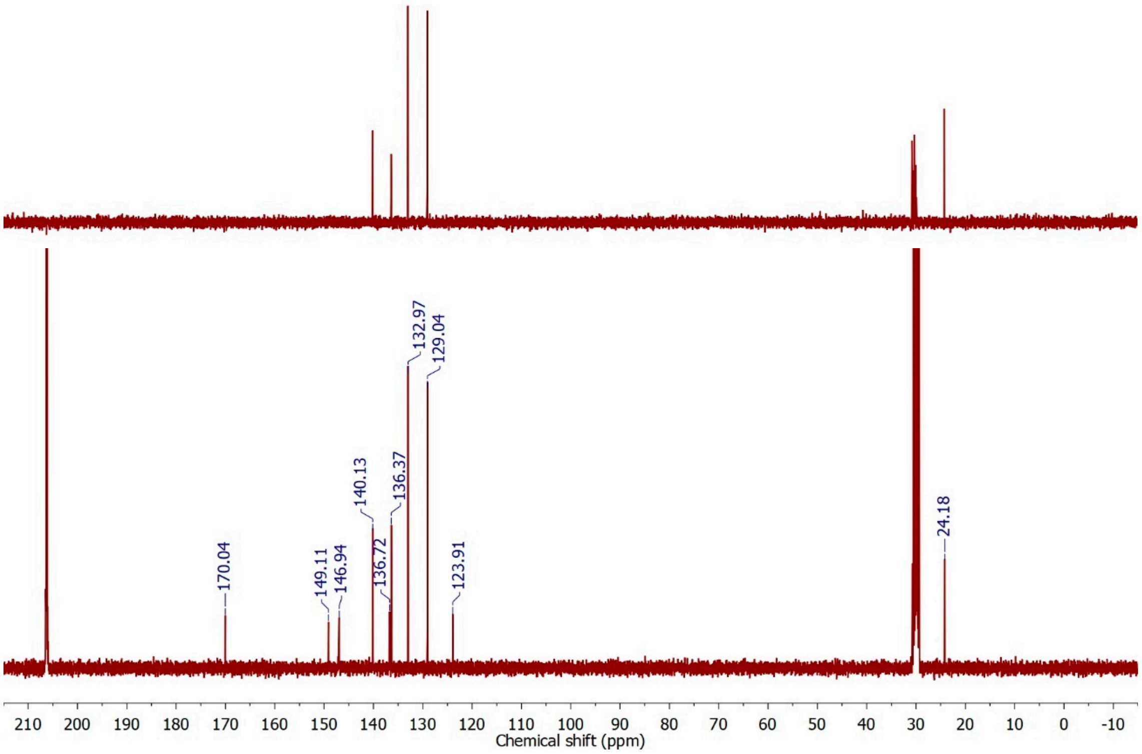

Figure S3. ^{1}H-NMR, DEPT, and ^{13}C-NMR spectra for **Br-Clmd**.

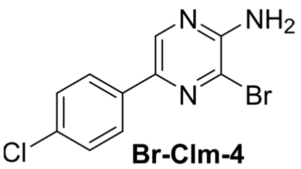

Br-Clm-4

^{1}H NMR (400 MHz, $CDCl_3$) δ = 8.38 – 8.35 (s, 1H), 7.83 – 7.79 (m, 2H), 7.43 – 7.39 (m, 2H), 5.27 – 5.18 (bs, 2H).

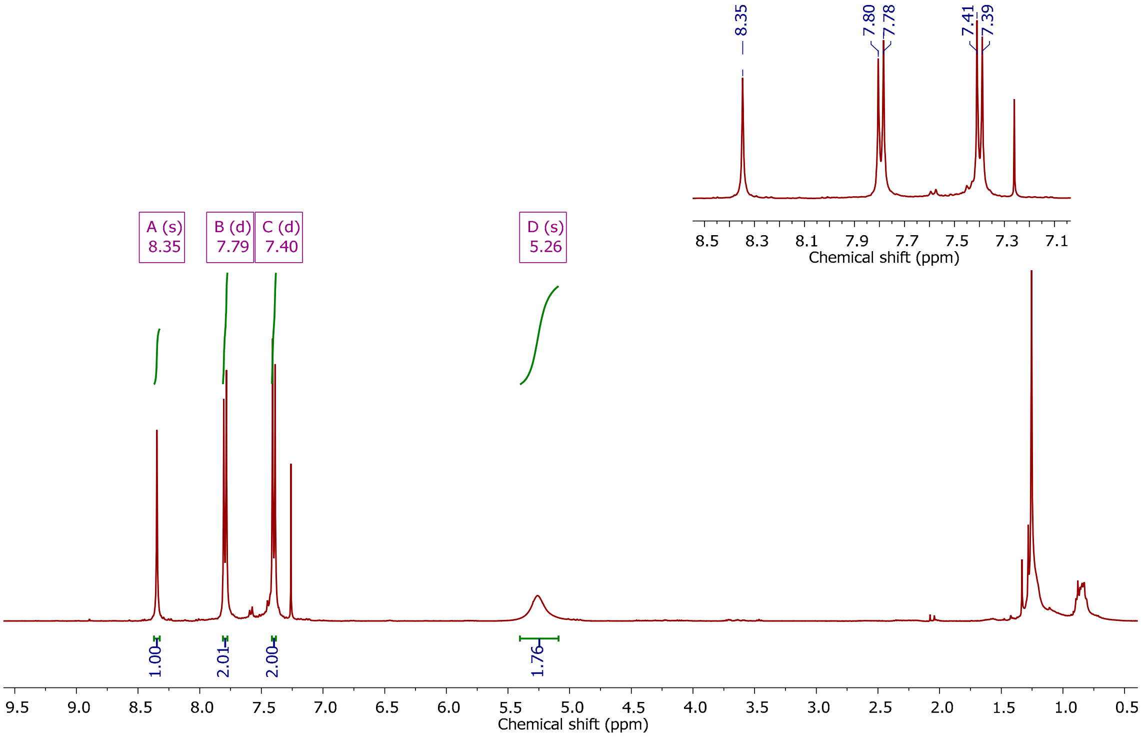

^{13}C NMR (101 MHz, $CDCl_3$) δ = 151.4 (C), 142.0 (C), 137.1 (CH), 134.8 (C), 134.0 (C), 129.2 (2xCH), 127.0 (2xCH), 126.2 (C).

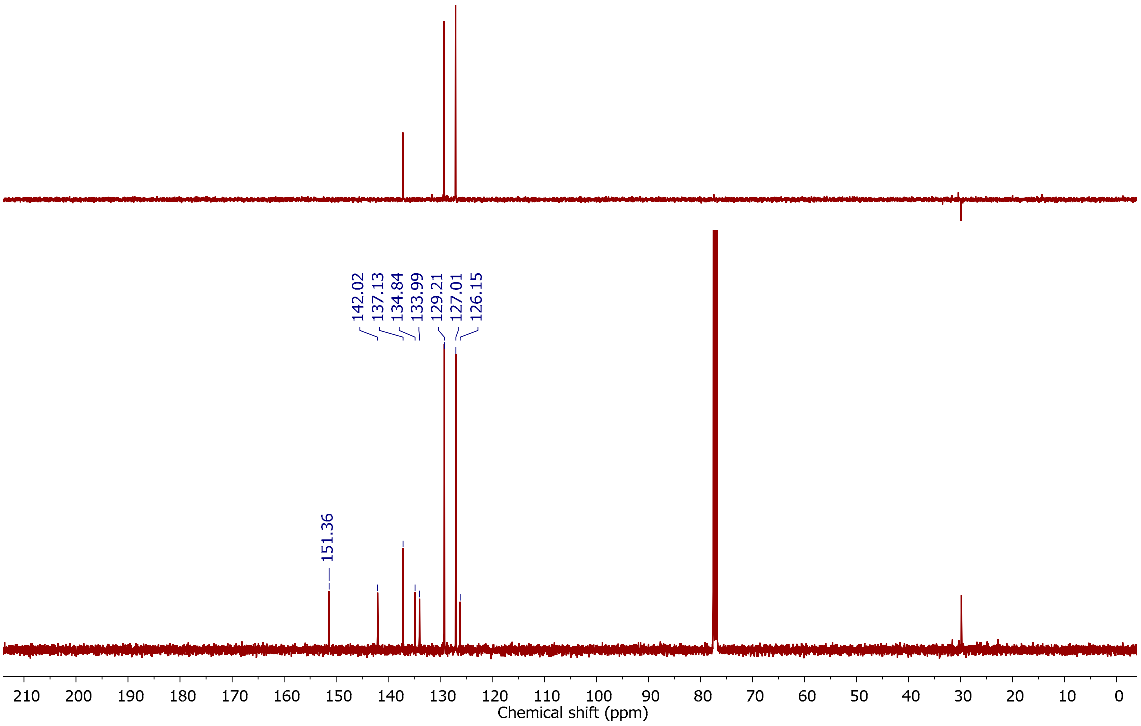

Figure S4. ^{1}H-NMR, DEPT, and ^{13}C-NMR spectra for **Br-Clm-4**.

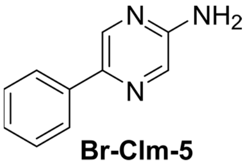

Br-Clm-5

^{1}H NMR (400 MHz, $CDCl_3$) δ = 8.49 – 8.42 (d, *J*=1.5 Hz, 1H), 8.12 – 8.05 (d, *J*=1.6, 1H), 7.90 – 7.85 (m, 2H), 7.48 – 7.41 (m, 2H), 7.39 – 7.34 (m, 1H), 4.82 – 4.56 (s, 2H).

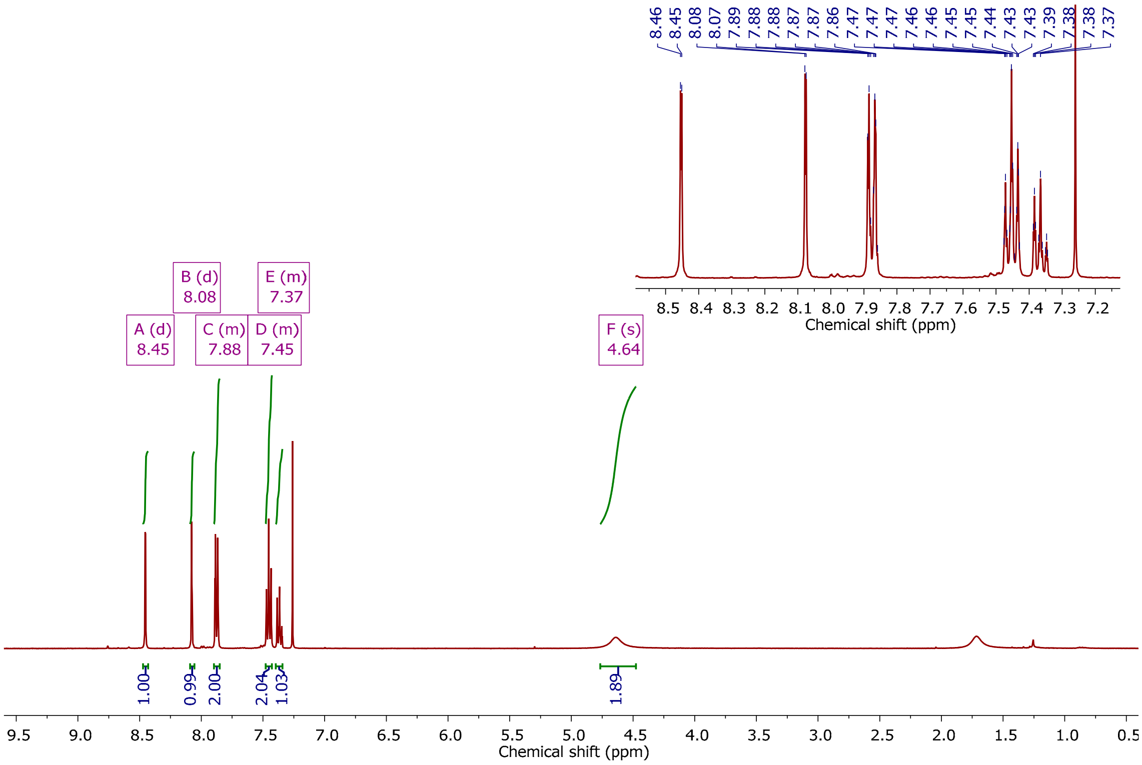

^{13}C NMR (101 MHz, $CDCl_3$) δ = 153.2 (C), 143.2 (C), 139.2 (CH), 137.1 (C), 131.8 (CH), 129.0 (2xCH), 128.3 (CH), 125.8 (2xCH).

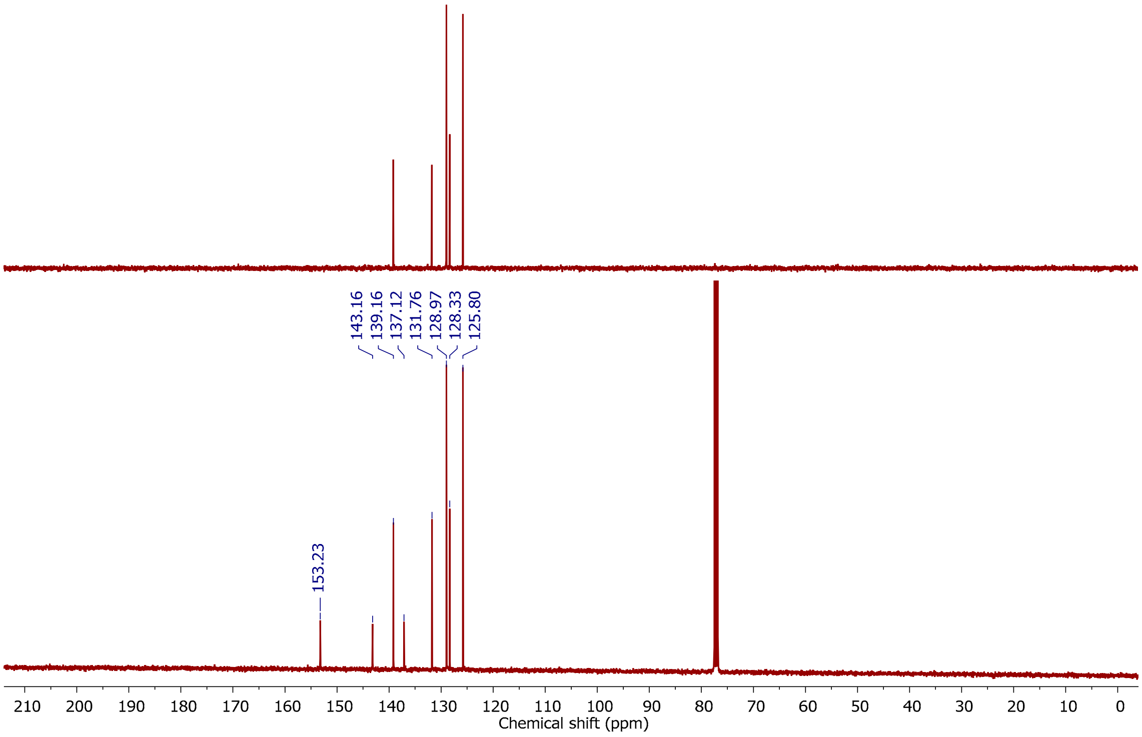

Figure S5. ^{1}H-NMR, DEPT, and ^{13}C-NMR spectra for **Br-Clm-5**.

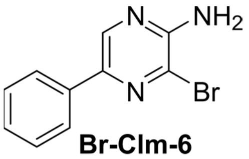

Br-Clm-6

^{1}H NMR (400 MHz, $CDCl_3$) δ = 8.52 – 8.29 (s, 1H), 7.91 – 7.80 (m, 2H), 7.47 – 7.41 (m, 2H), 7.41 – 7.31 (m, 1H), 5.17 (bs, 2H).

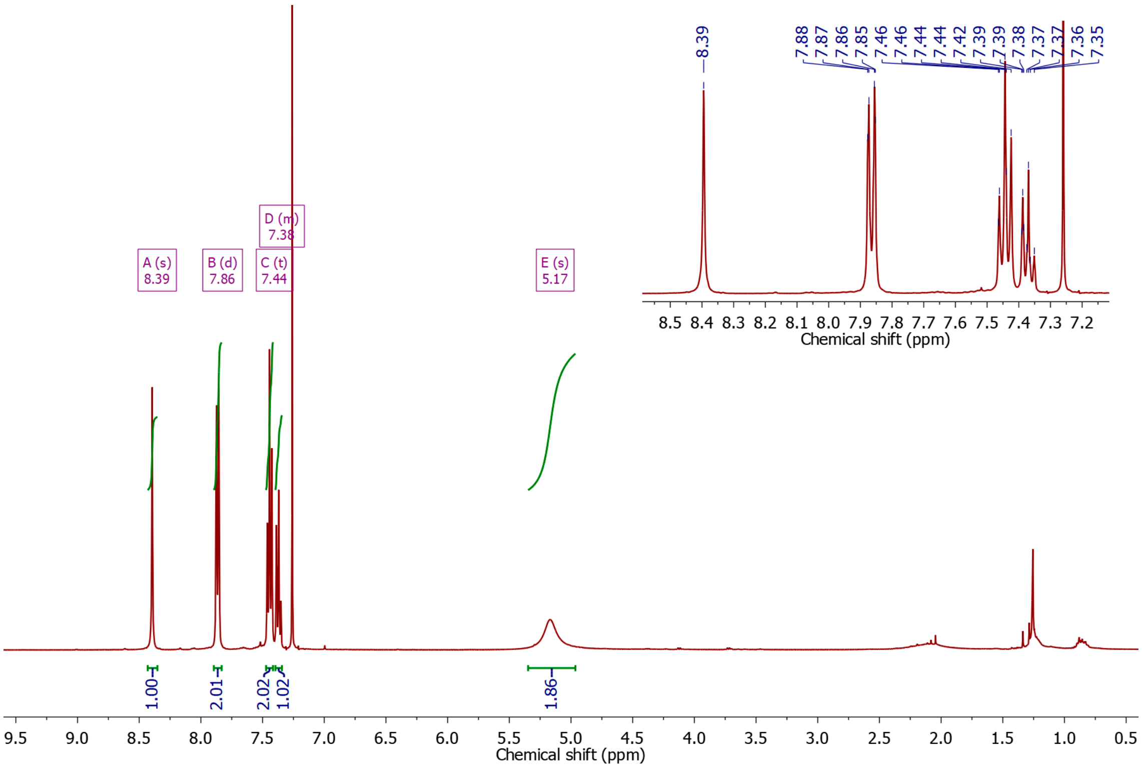

^{13}C NMR (101 MHz, $CDCl_3$) δ = 151.3 (CH), 143.4 (C), 137.7 (C), 135.6 (C), 129.0 (CH), 128.8 (CH), 126.0 (C), 125.9 (CH).

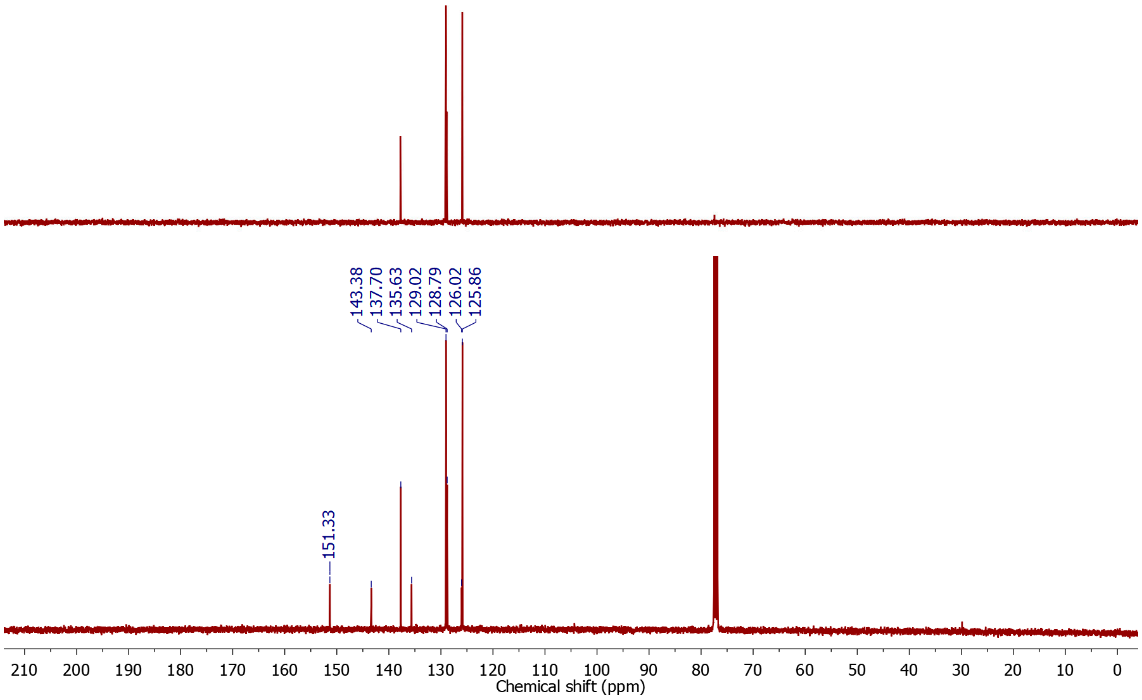

Figure S6. ^{1}H-NMR, DEPT, and ^{13}C-NMR spectra for **Br-Clm-6**.

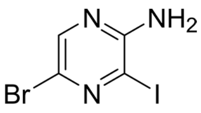

5-Bromo-3-iodo-pyrazin-2-amine

^{1}H NMR (400 MHz, $CDCl_3$) δ = 7.97 (s, 1H), 5.07 (bs, 2H).

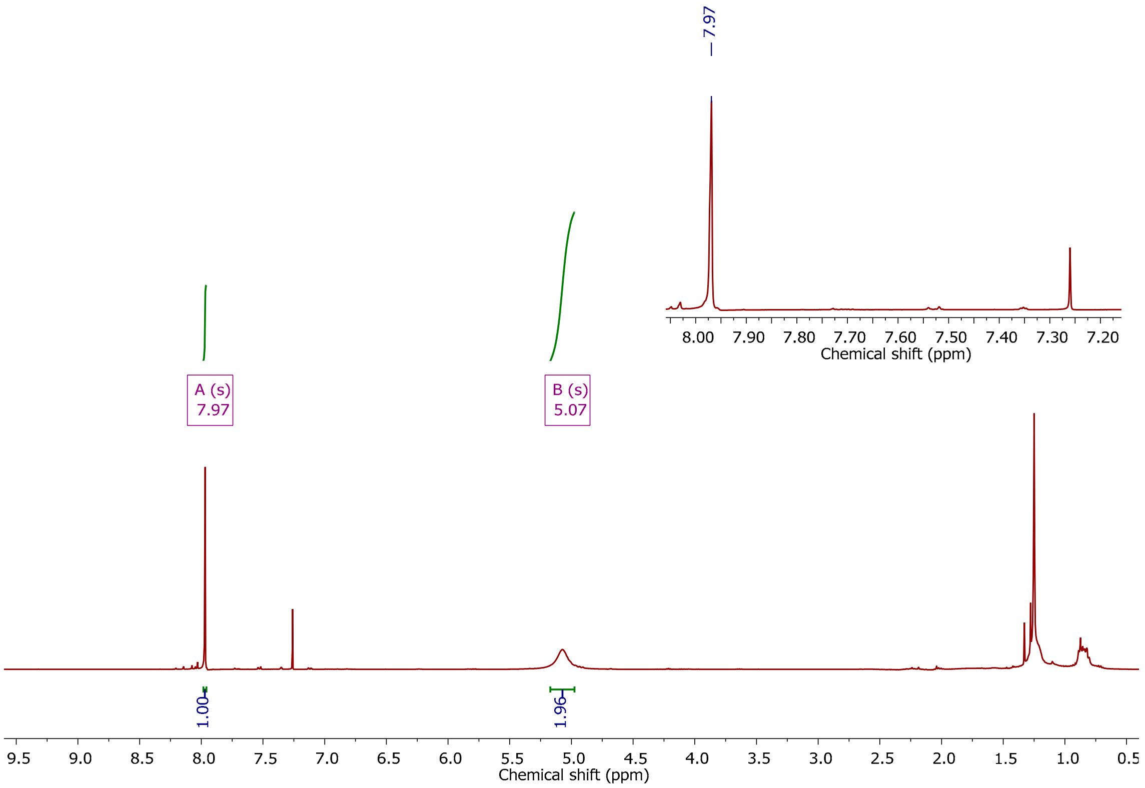

^{13}C NMR (101 MHz, $CDCl_3$) δ = 154.3 (C), 143.3 (CH), 124.5 (C), 101.8 (C).

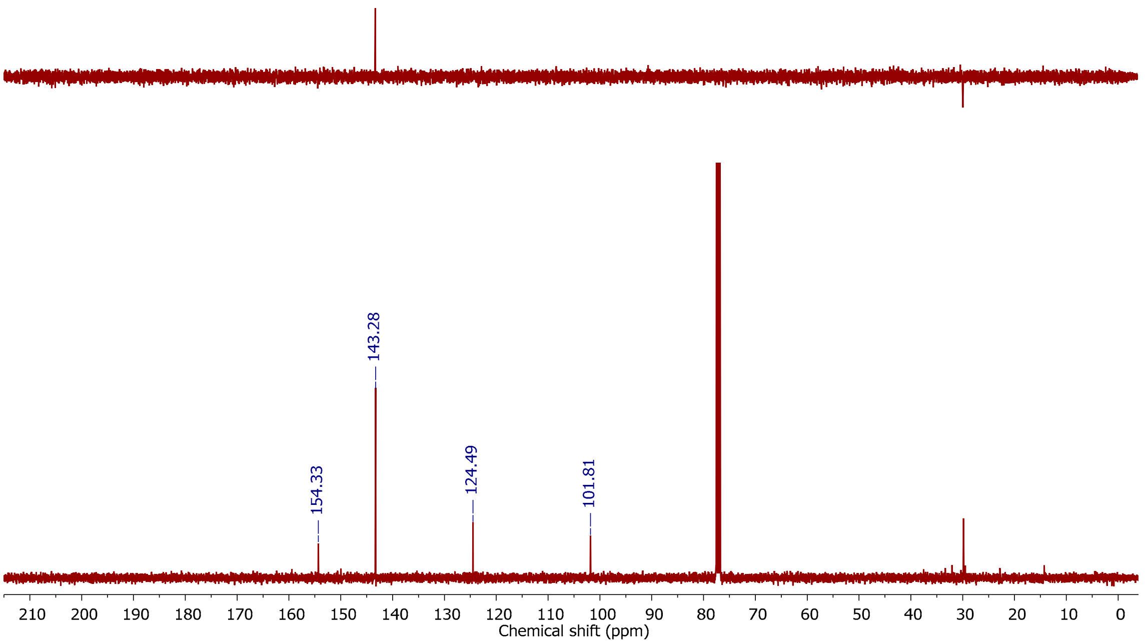

Figure S7. ^{1}H-NMR, DEPT, and ^{13}C-NMR spectra for 5-bromo-3-iodopyrazin-2-amine.

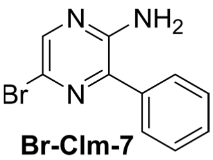

^{1}H NMR (400 MHz, $CDCl_3$) δ = 8.08 – 8.03 (s, 1H), 7.76 – 7.68 (m, 2H), 7.54 – 7.43 (m, 3H), 4.90 (bs, 2H).

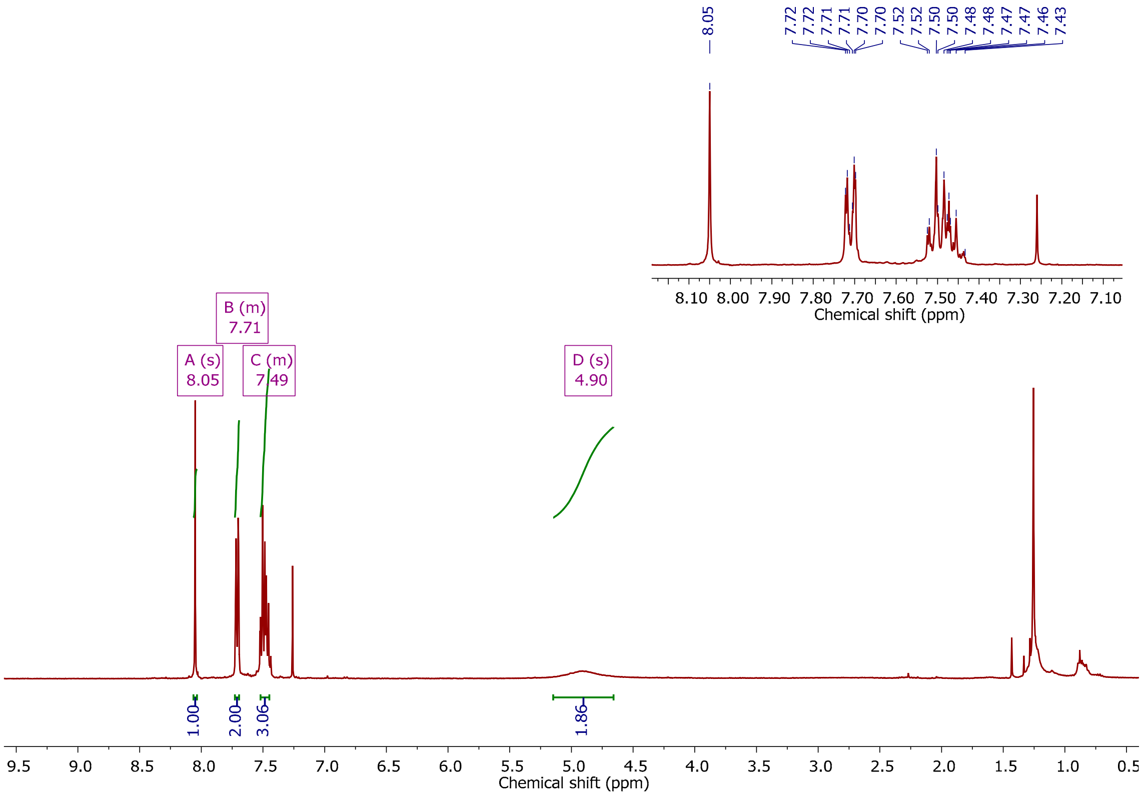

^{13}C NMR (101 MHz, $CDCl_3$) δ = 151.1 (C), 142.1 (CH), 141.3 (C), 135.8 (C), 129.9 (CH), 129.4 (2xCH), 128.2 (2xCH), 127.0 (C).

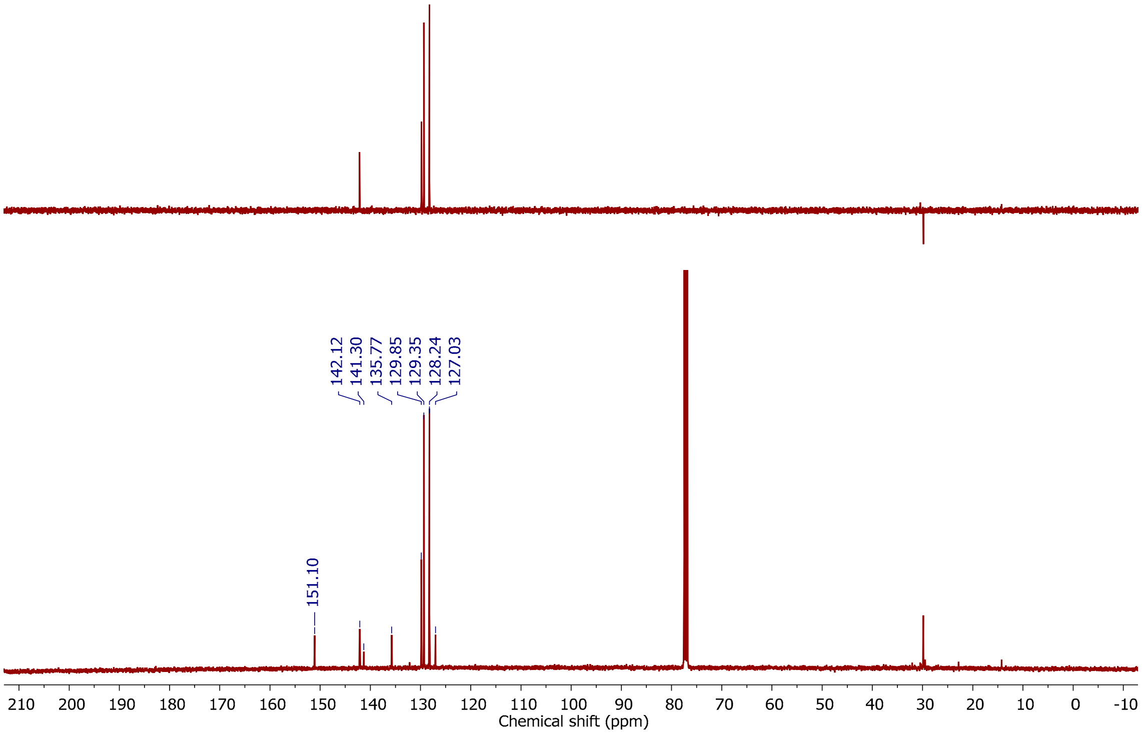

Figure S8. ^{1}H-NMR, DEPT, and ^{13}C-NMR spectra for **Br-Clm-7**.

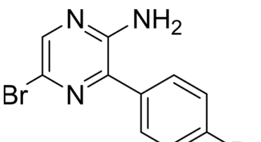

Br-Clm-8

^{1}H NMR (400 MHz, $CDCl_3$) δ = 8.08 – 8.07 (s, 1H), 7.68 – 7.58 (m, 4H), 4.94 (bs, 2H).

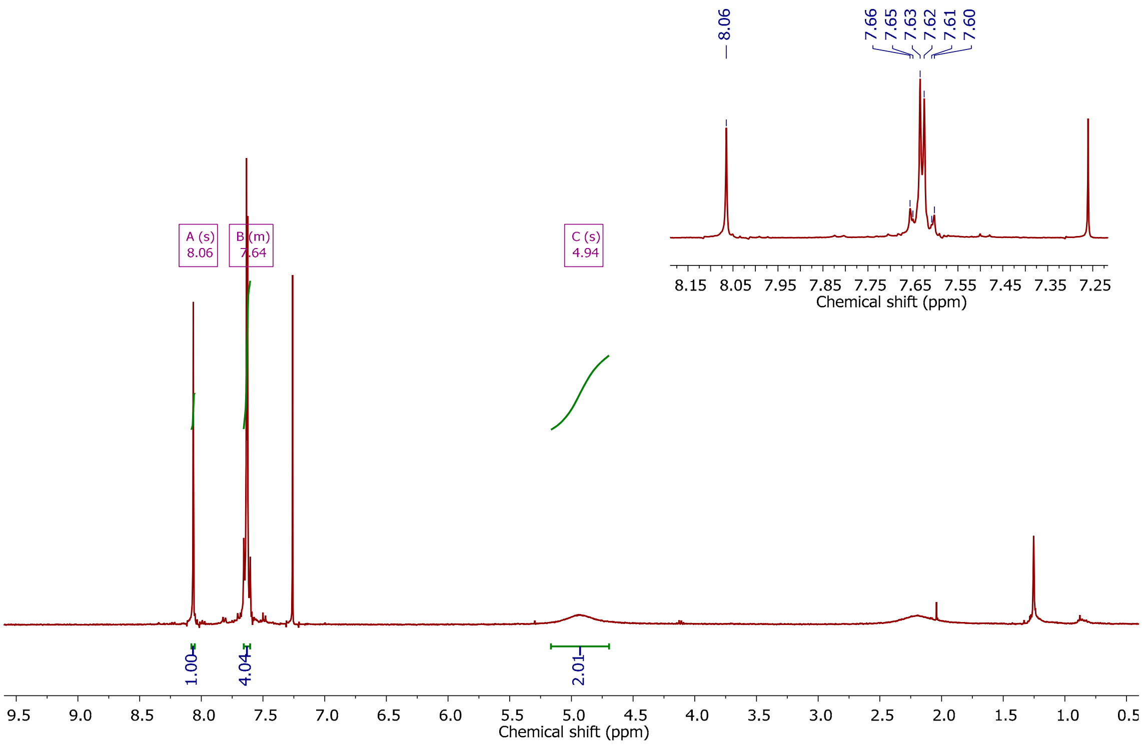

^{13}C NMR (101 MHz, $CDCl_3$) δ = 150.9 (C), 142.3 (CH), 140.1 (C), 134.6 (C), 132.6 (2xCH), 129.9 (2xCH), 127.2 (C), 124.3 (C).

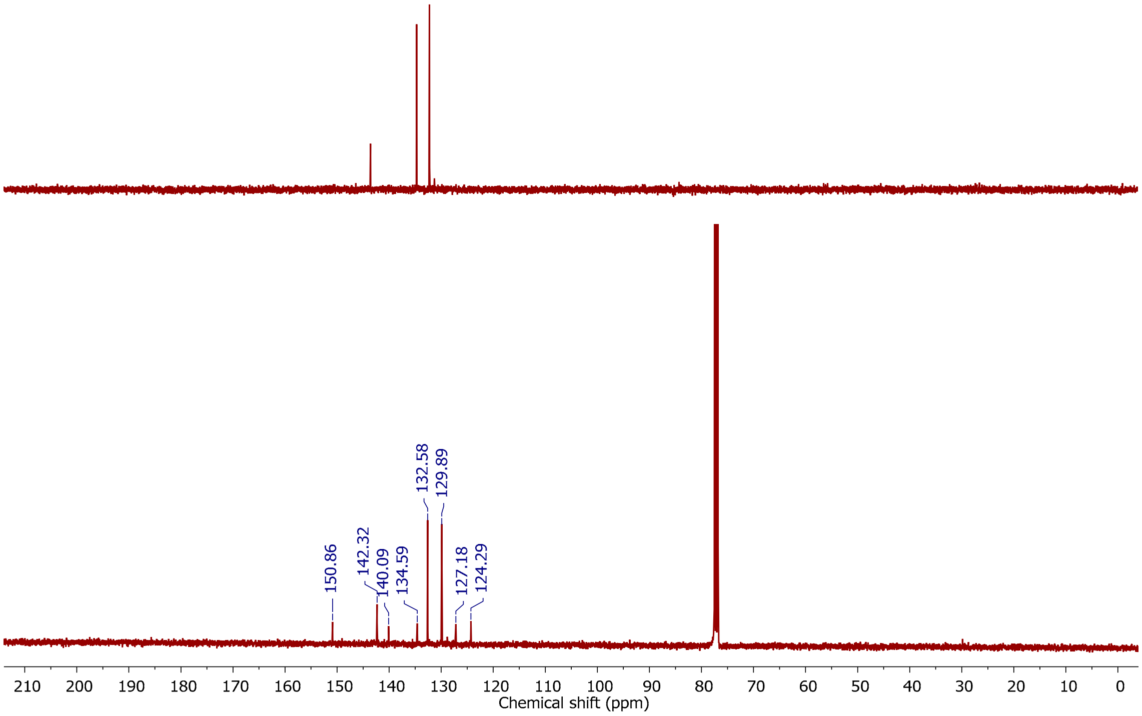

Figure S9. ^{1}H-NMR, DEPT, and ^{13}C-NMR spectra for **Br-Clm-8**.

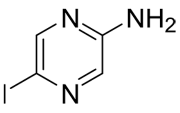

Br-Clm-11

^{1}H NMR (400 MHz, $CDCl_3$) δ = 8.44 – 8.15 (d, *J*=1.5, 1H), 8.05 – 7.70 (d, *J*=1.4, 1H), 4.64 – 4.55 (s, 3H).

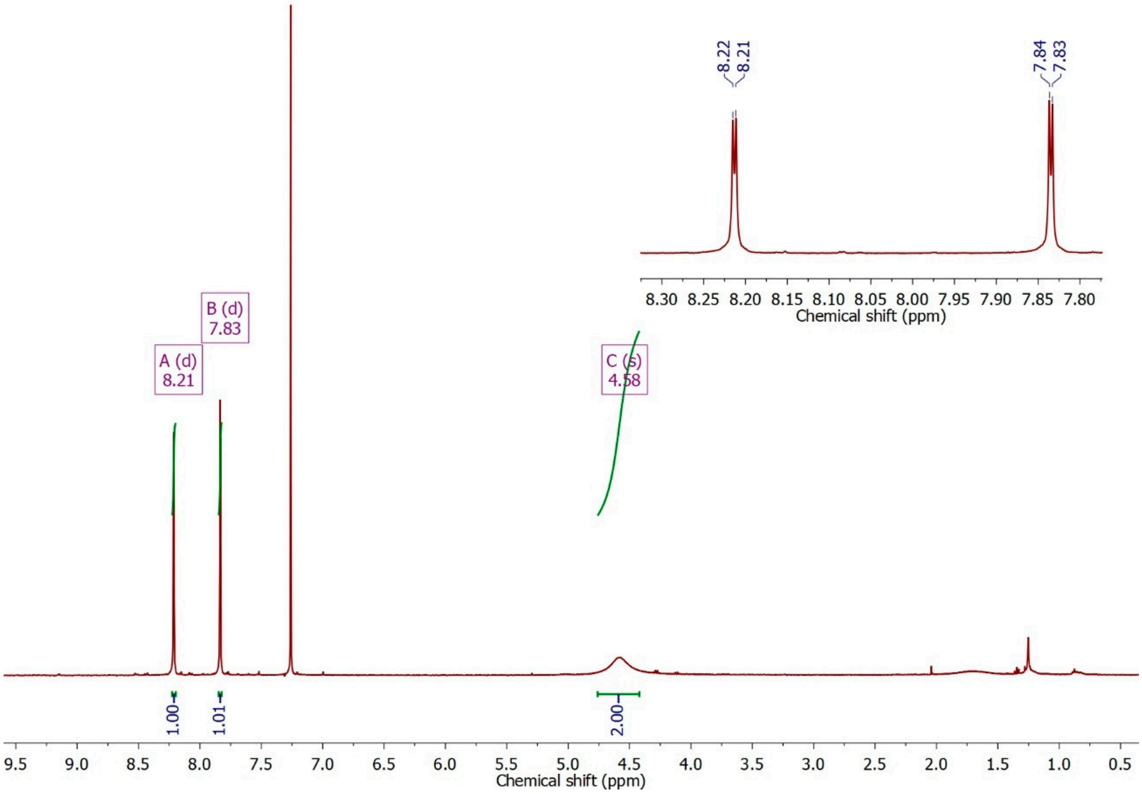

^{13}C NMR (101 MHz, $CDCl_3$) δ = 153.7 (C), 149.7 (CH), 133.6 (CH), 100.6 (C).

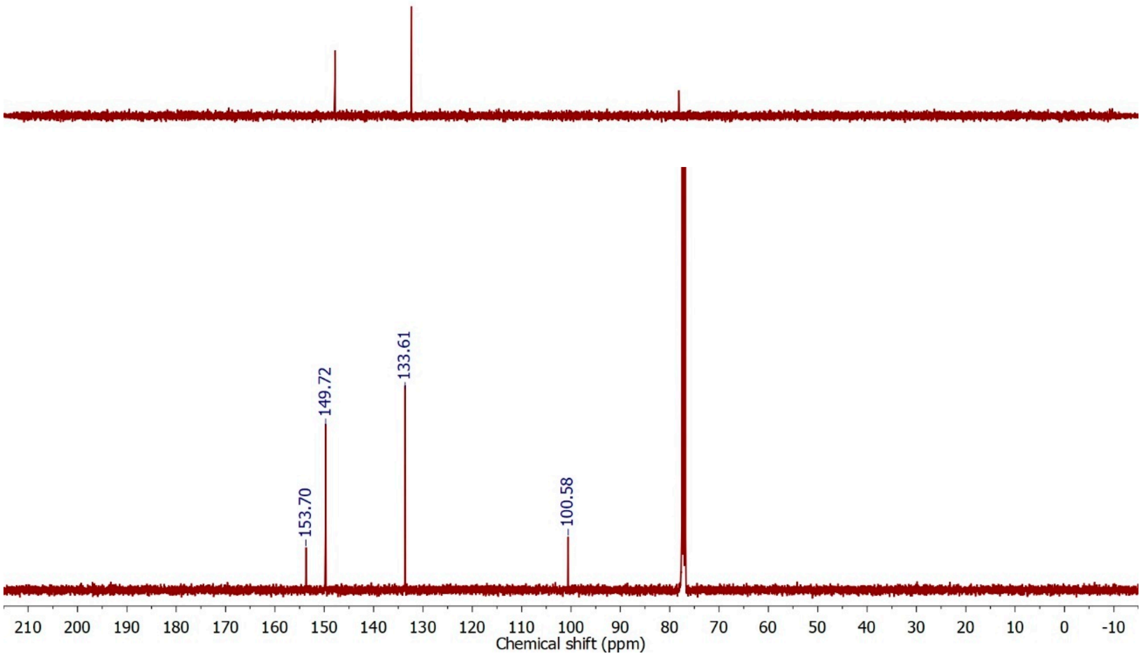

Figure S10. ^{1}H-NMR, DEPT, and ^{13}C-NMR spectra for **Br-Clm-11**.

2.2. FT-MS Spectra

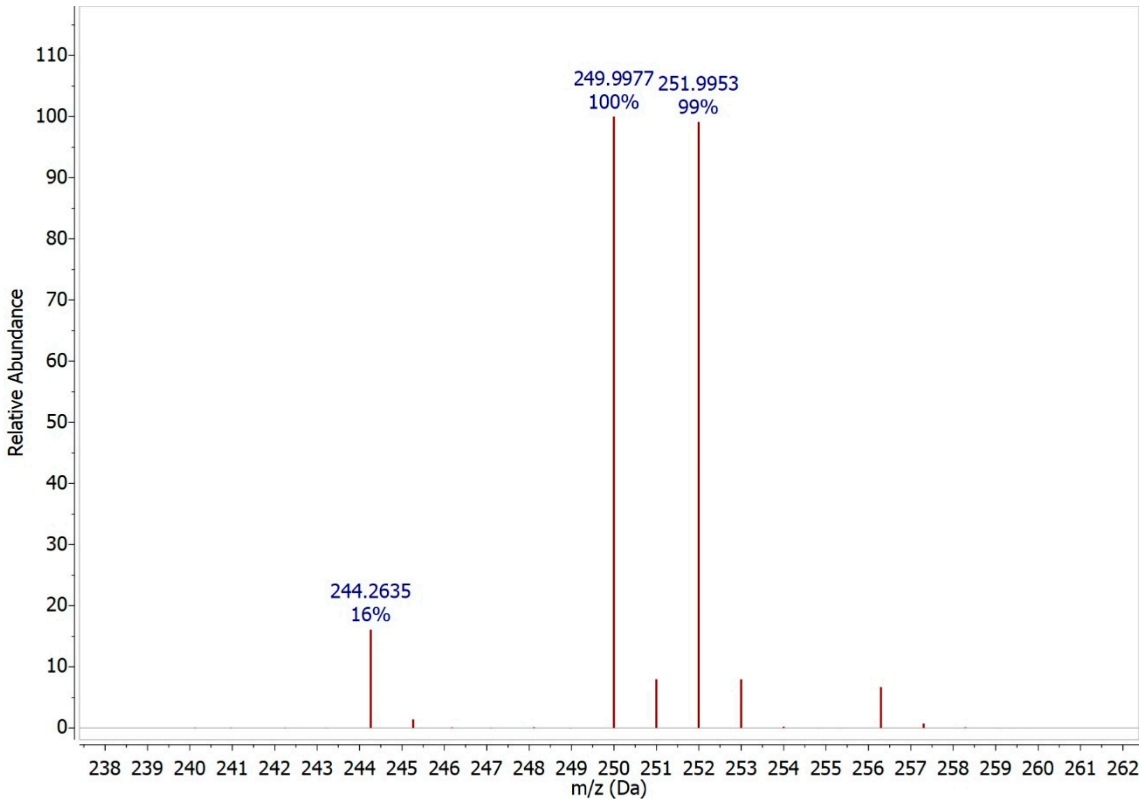

Figure S11. FTMS-ESI (+) spectrum for **Br-Clm-1**: m/z: calcd for $[C_{10}H_9BrN_3]^+$: 249.9980 [M+H]$^+$; found 249.9977 $[C_{10}H_9{}^{79}BrN_3]^+$, 251.9953 $[C_{10}H_9{}^{81}BrN_3]^+$.

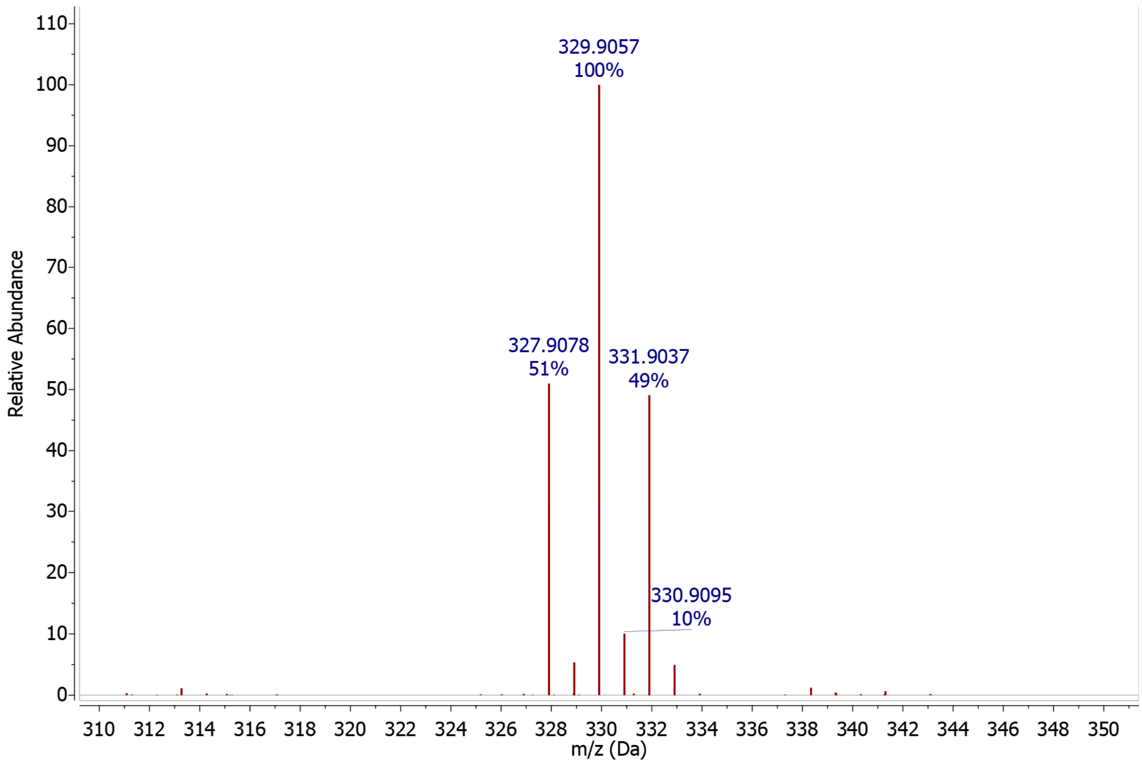

Figure S12. FTMS-ESI (+) spectrum for **Br-Clm-2**: m/z: calcd for $[C_{10}H_8Br_2N_3]^+$: 327.9085 [M+H]$^+$; found 327.9079 $[C_{10}H_8{}^{79}Br_2N_3]^+$, 329.9057 $[C_{10}H_8{}^{81}Br_2N_3]^+$, 331.9037 $[C_{10}H_8{}^{79}Br^{81}BrN_3]^+$.

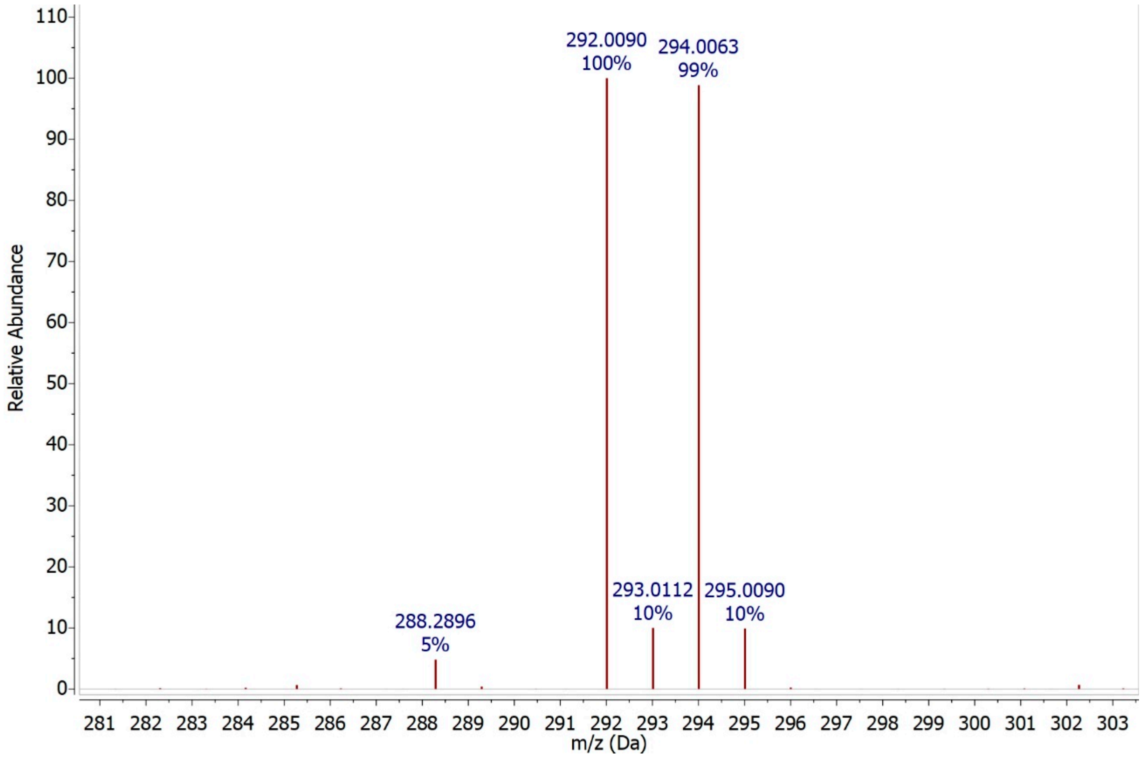

Figure S13. FTMS-ESI (+) spectrum for **Br-Clmd**: m/z: calcd for $[C_{12}H_{11}BrN_3O]^+$: 292.0085 $[M+H]^+$; found 292.0090 $[C_{12}H_{11}{}^{79}BrN_3O]^+$, 294.0063 $[C_{12}H_{11}{}^{81}BrN_3O]^+$.

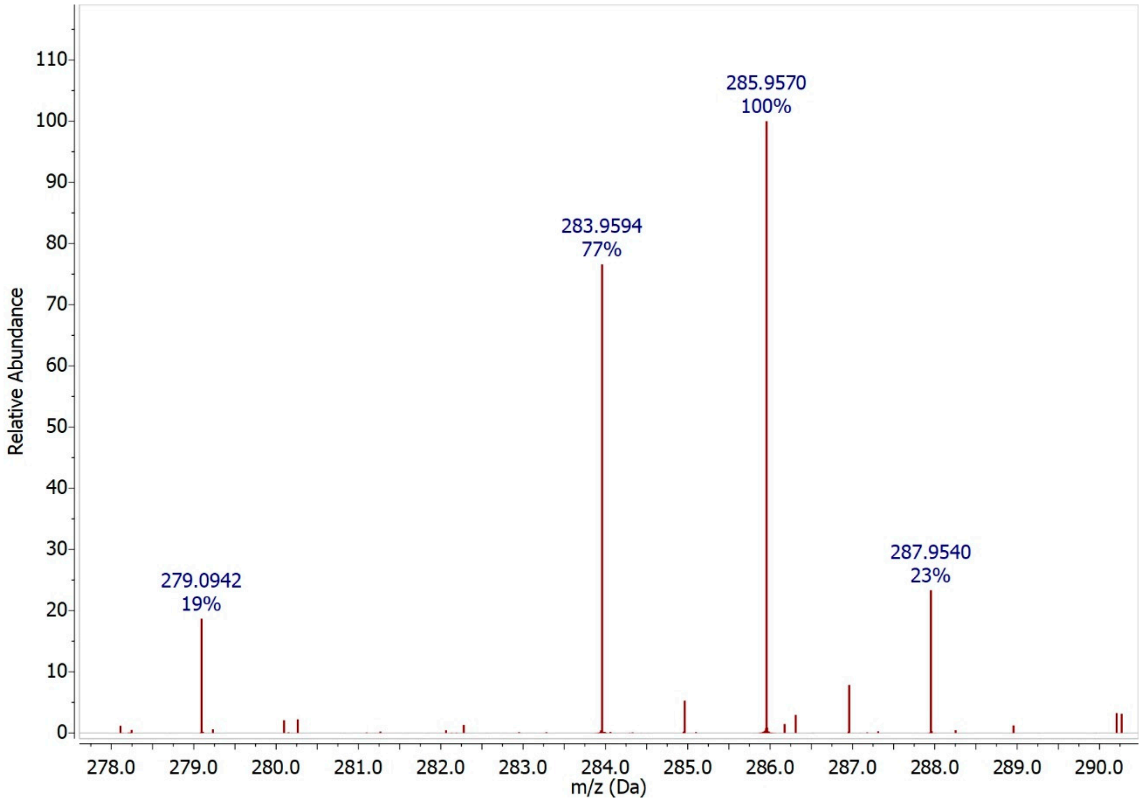

Figure S14. FTMS-ESI (+) spectrum for **Br-Clm-4**: m/z: calcd for $[C_{10}H_8BrClN_3]^+$: 283.9590 $[M+H]^+$; found 283.9591 $[C_{10}H_8{}^{79}Br^{35}ClN_3]^+$, 285.9570 $[C_{10}H_8{}^{79}Br^{37}ClN_3]^+$, and $[C_{10}H_8{}^{81}Br^{35}ClN_3]^+$, 287.9510 $[C_{10}H_8{}^{81}Br^{37}ClN_3]^+$.

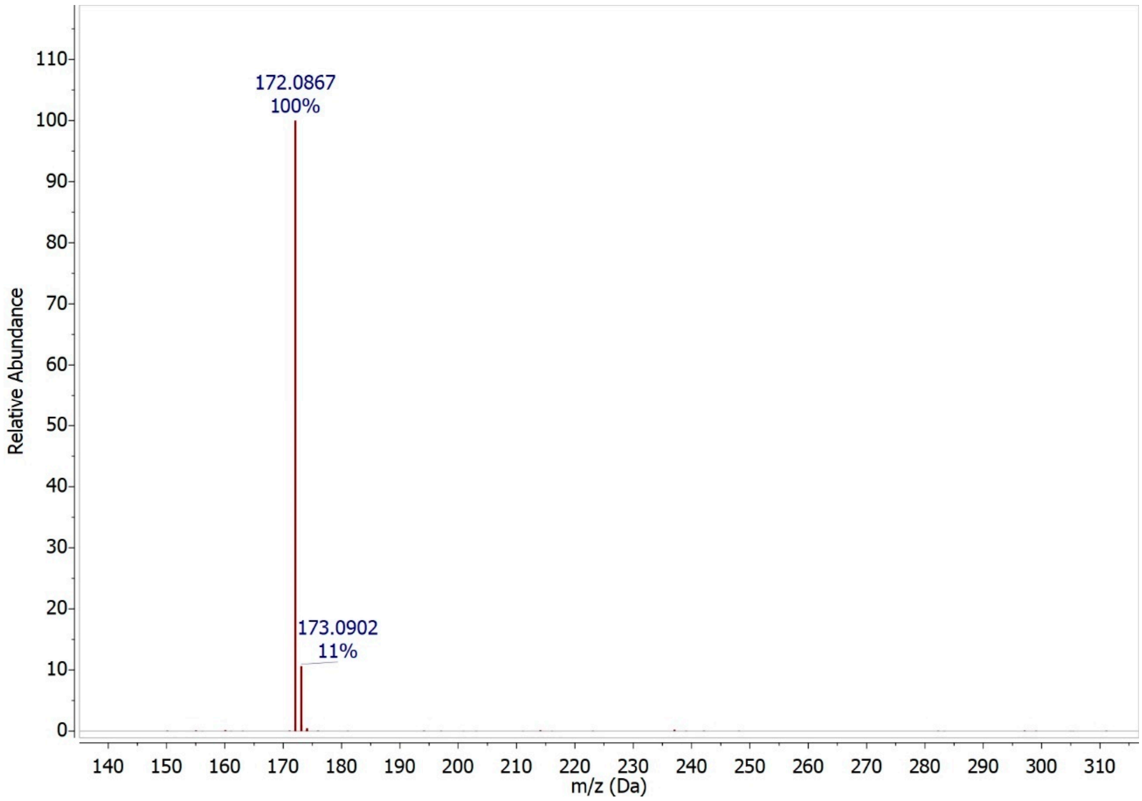

Figure S15. FTMS-ESI (+) spectrum for **Br-Clm-5**: m/z: calcd for $[C_{10}H_{10}N_3]^+$: 172.0875 [M+H]$^+$; found 172.0867 $[C_{10}H_{10}N_3]^+$.

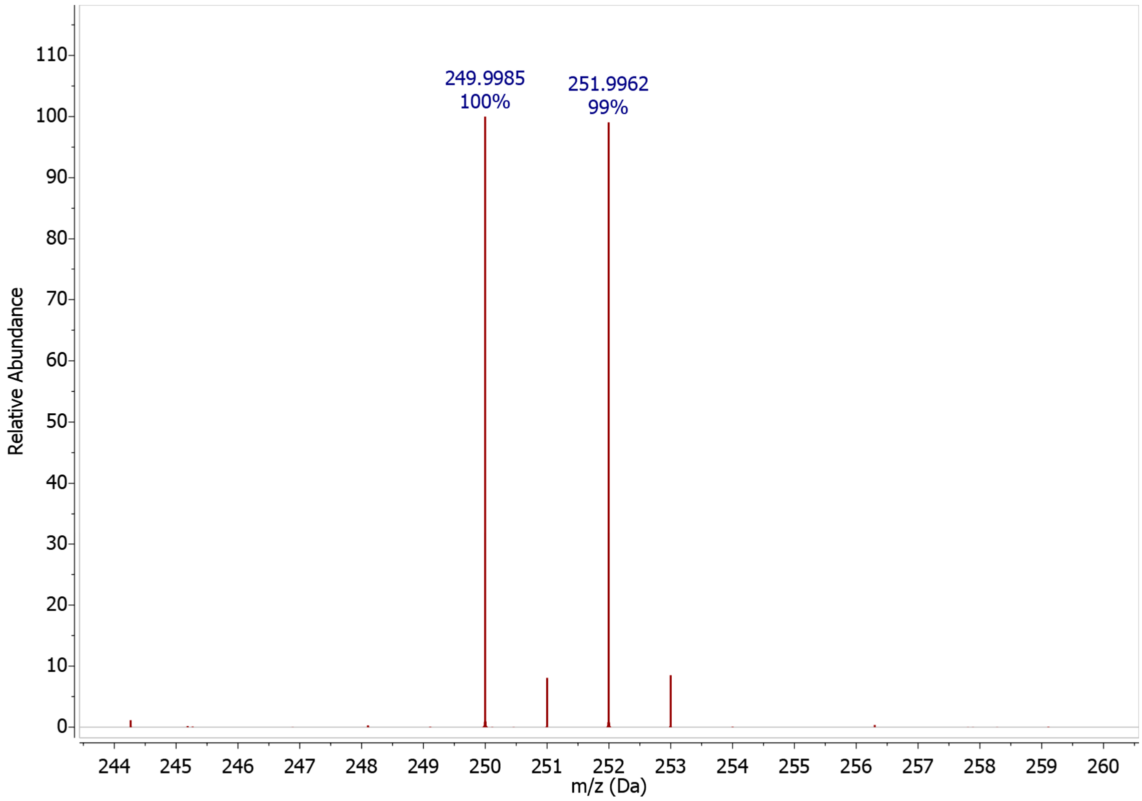

Figure S16. FTMS-ESI (+) spectrum for **Br-Clm-6**: m/z: calcd for $[C_{10}H_9BrN_3]^+$: 249.9980 [M+H]$^+$; found 249.9985 $[C_{10}H_9{}^{79}BrN_3]^+$, 251.9962 $[C_{10}H_9{}^{81}BrN_3]^+$.

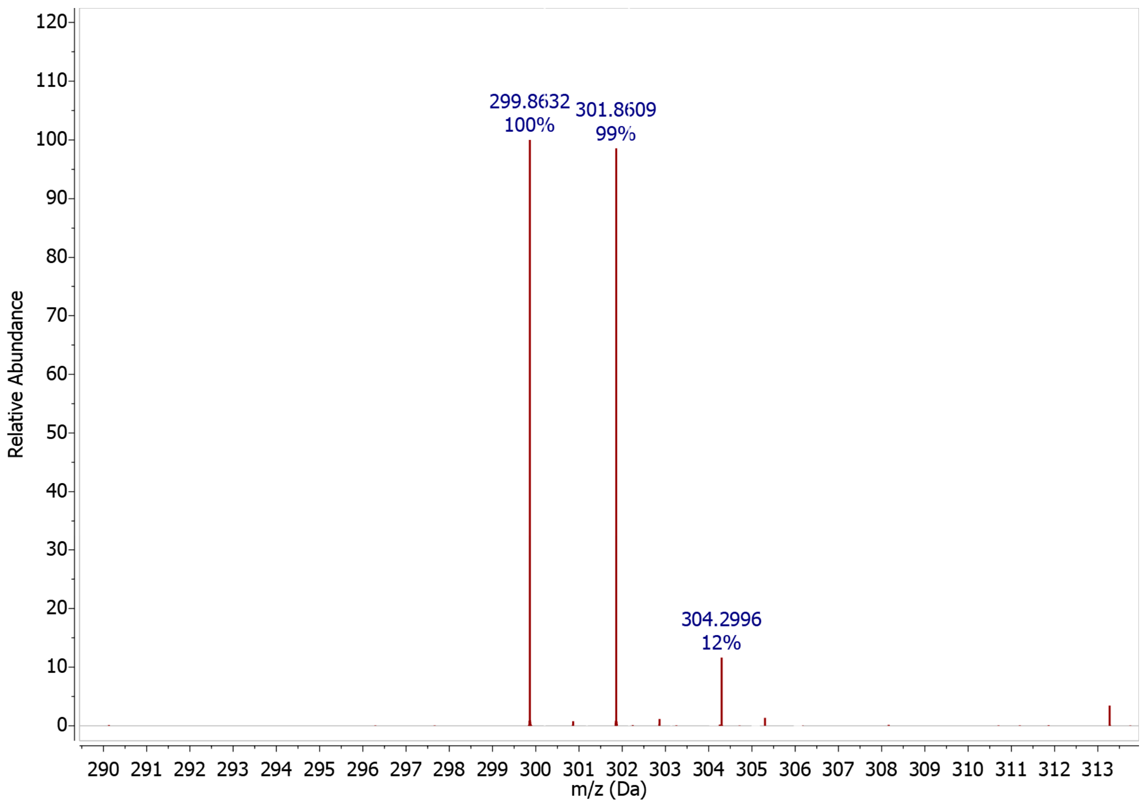

Figure S17. FTMS-ESI (+) spectrum for 5-bromo-3-iodopyrazin-2-amine: m/z: calcd for $[C_4H_4BrIN_3]^+$: 299.8633 $[M+H]^+$; found 299.8632 $[C_4H_4{}^{79}BrIN_3]^+$, 301.8609 $[C_4H_4{}^{81}BrIN_3]^+$.

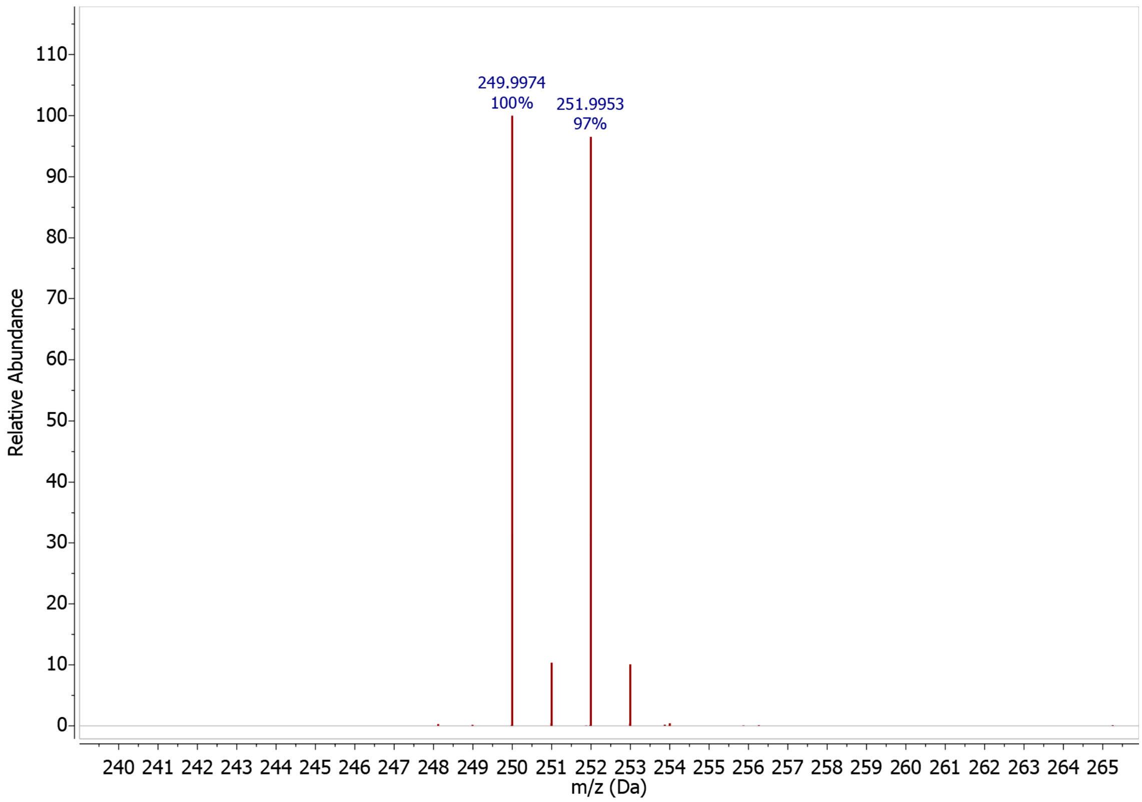

Figure S18. FTMS-ESI (+) spectrum for **Br-Clm-7**: m/z: calcd for $[C_{10}H_9BrN_3]^+$: 249.9980 $[M+H]^+$; found 249.9974 $[C_{10}H_9{}^{79}BrN_3]^+$, 251.9953 $[C_{10}H_9{}^{81}BrN_3]^+$.

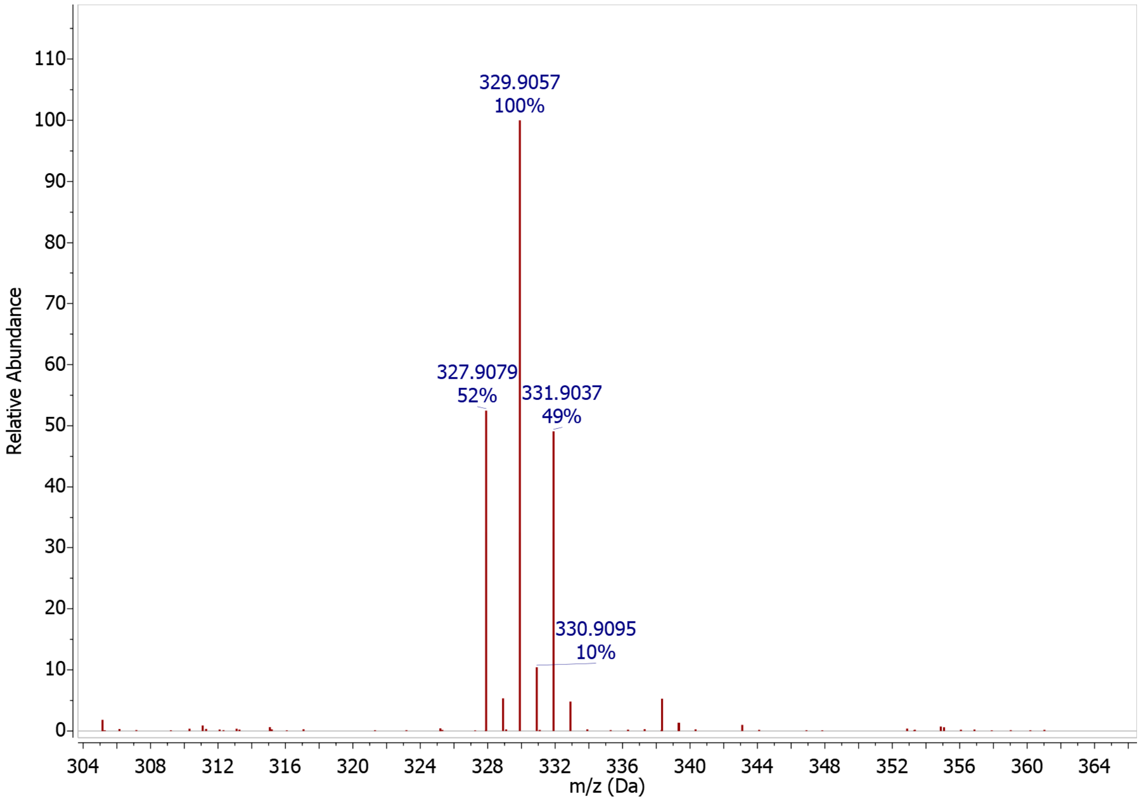

Figure S19. FTMS-ESI (+) spectrum for **Br-Clm-8**: m/z: calcd for $[C_{10}H_8Br_2N_3]^+$: 327.9085 $[M+H]^+$; found 327.9079 $[C_{10}H_8{}^{79}Br_2N_3]^+$, 329.9057 $[C_{10}H_8{}^{79}Br^{81}BrN_3]^+$, 331.9037 $[C_{10}H_8{}^{81}Br_2N_3]^+$.

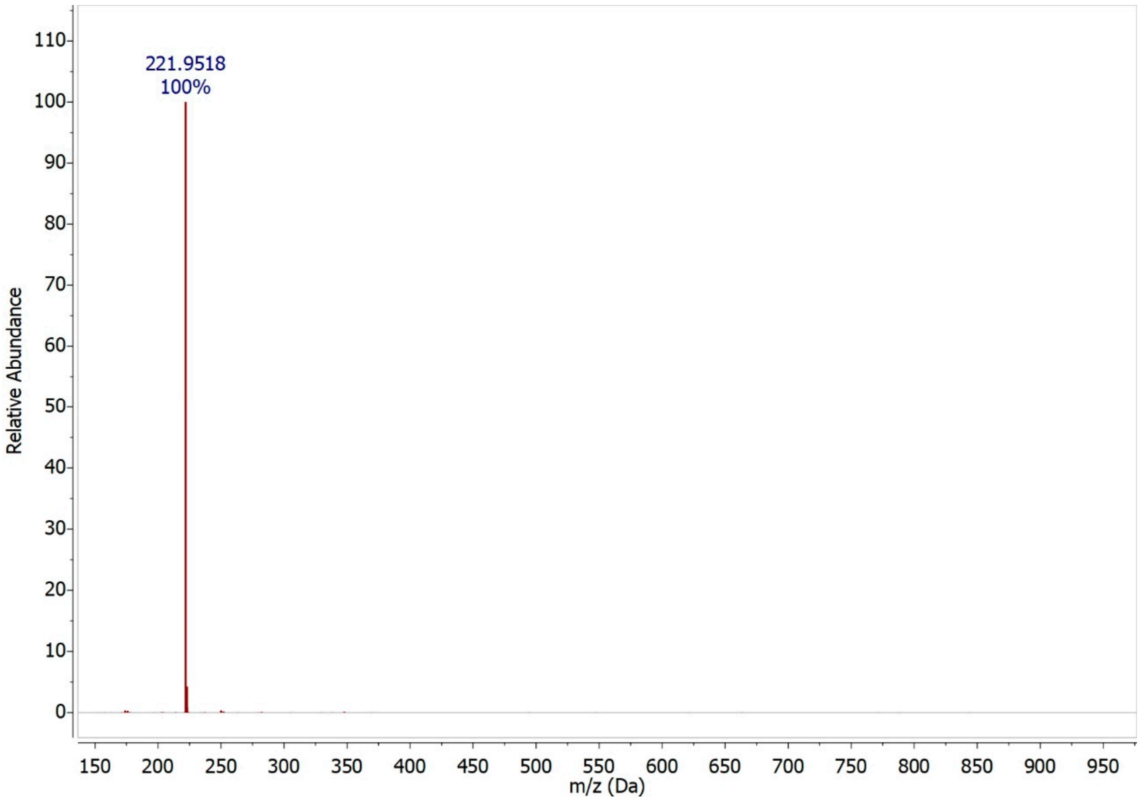

Figure S20. FTMS-ESI (+) spectrum for **Br-Clm-11**: m/z: calcd for $[C_4H_5IN_3]^+$: 221.9528 $[M+H]^+$; found 221.9518.

3. Photophysical characterization

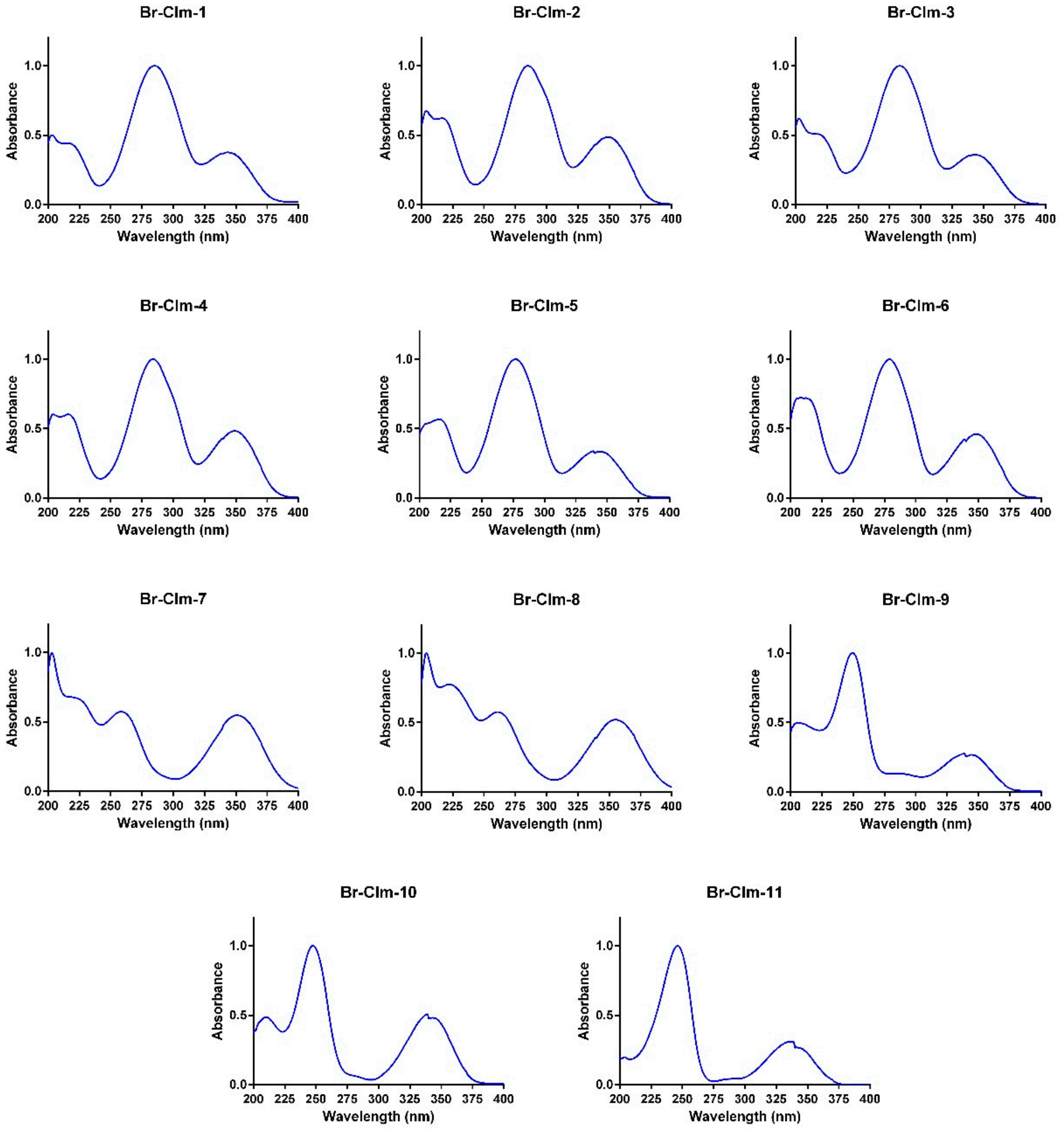

Figure S21. Absorbance spectra of the **Br-Clm** compounds in 30 μM methanolic solutions.

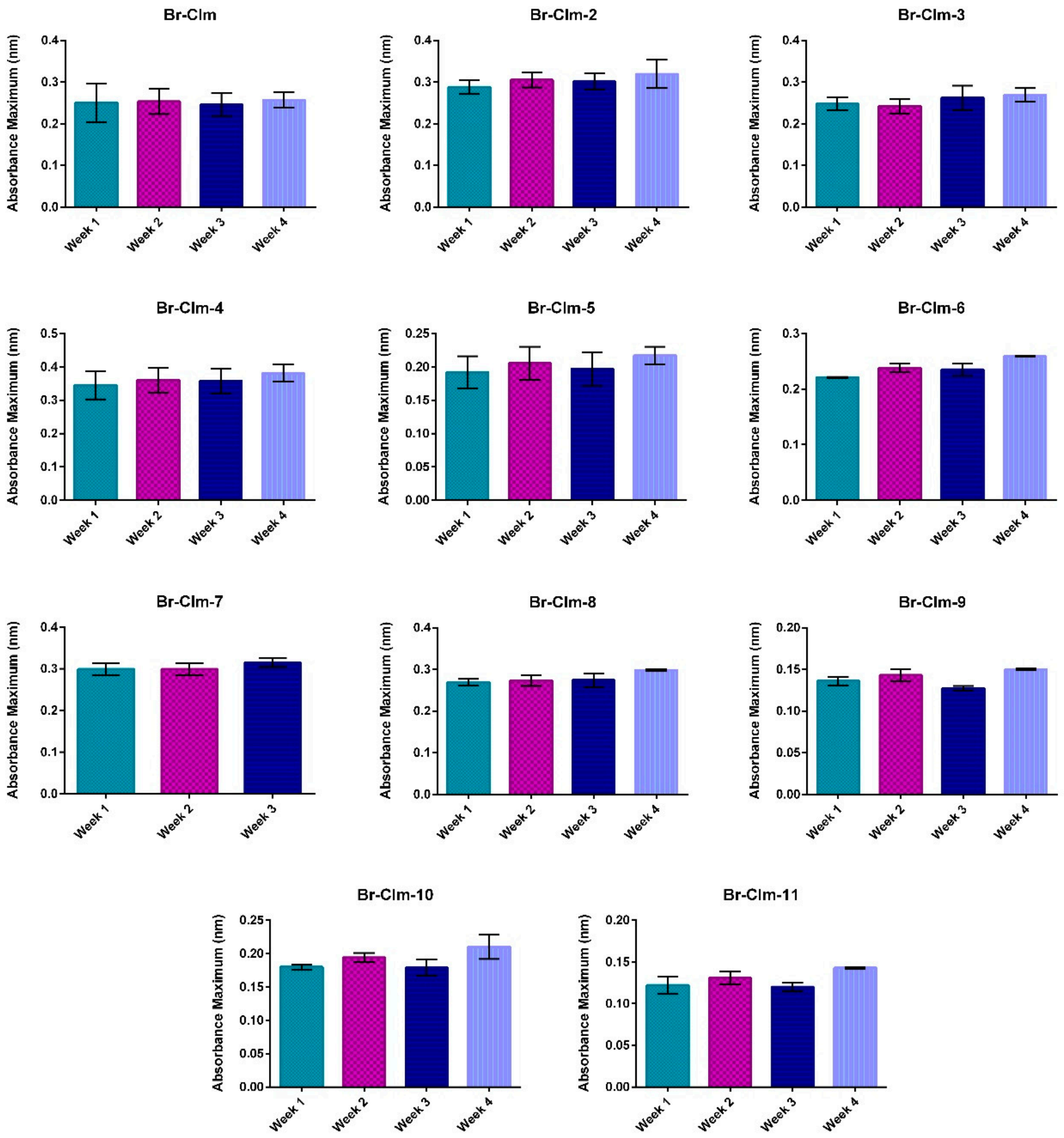

Figure S22. Maximum absorbance intensity of 30 μM methanolic solutions of the **Br-Clm** family, measured weekly. **Br-Clm-7** was only measured three times.

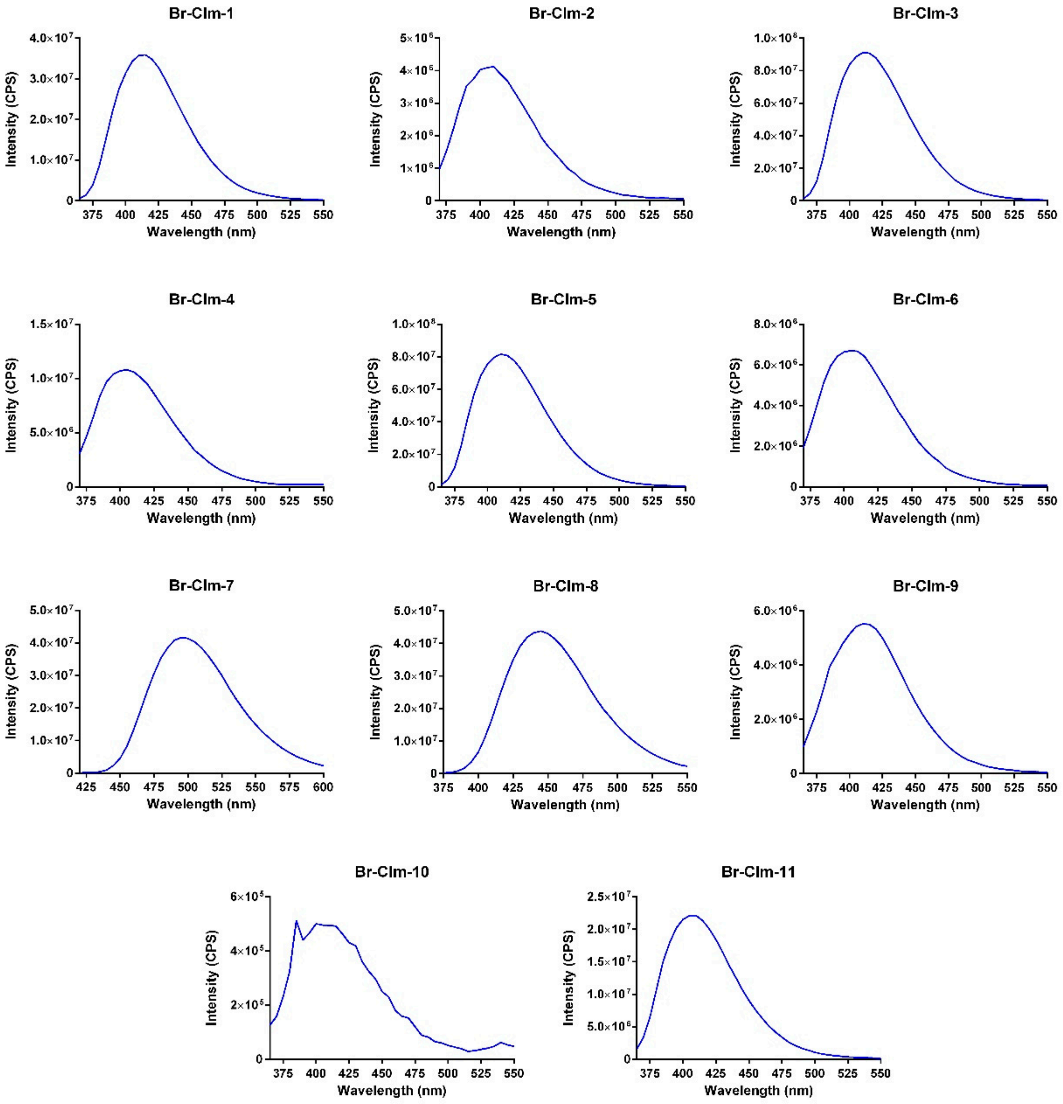

Figure S23. Emission spectra of the **Br-Clm** compounds in 30 μM methanolic solutions.

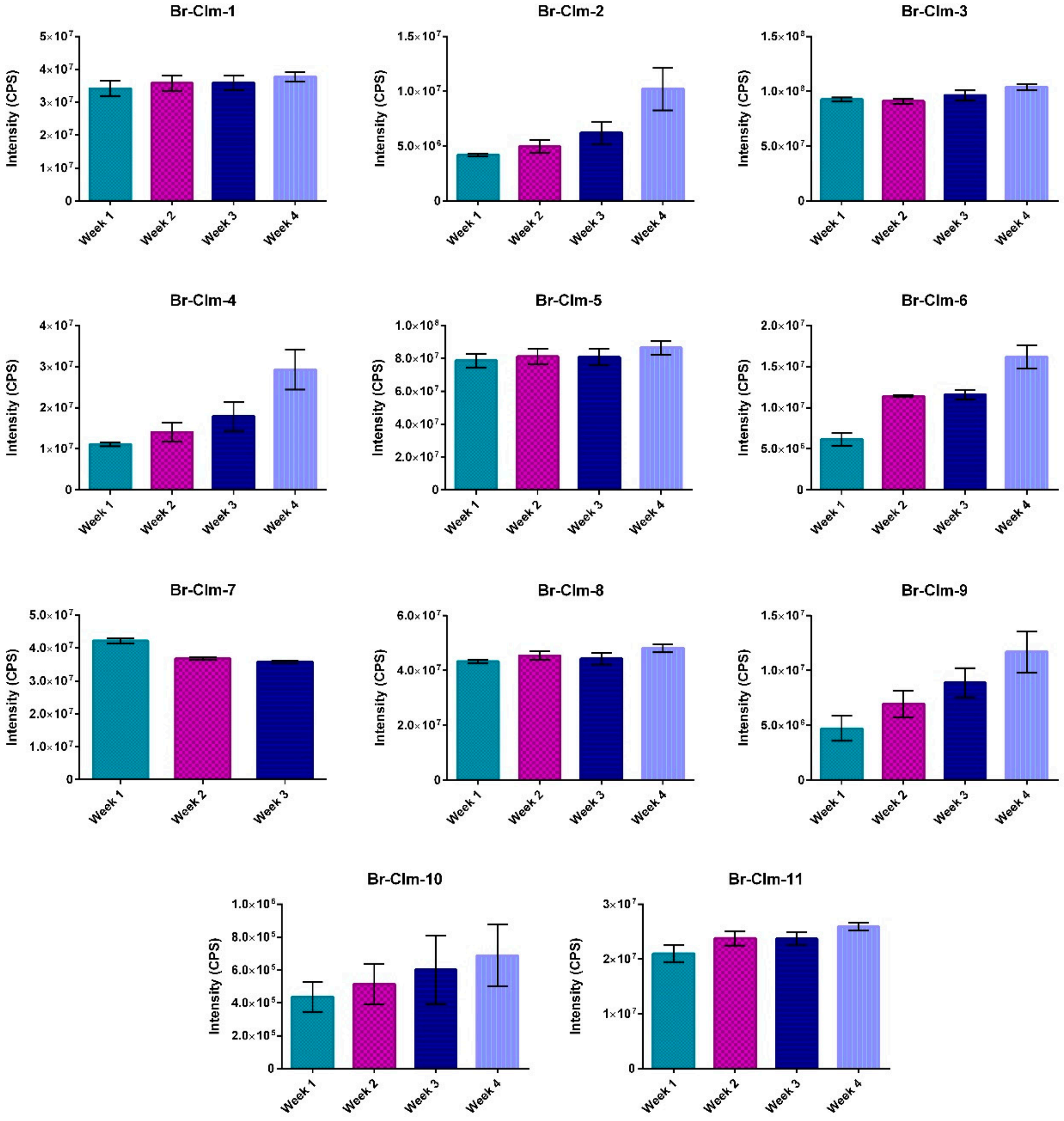

Figure S24. Maximum emission intensity of 30 μM methanolic solutions of the **Br-Clm** family, measured weekly. **Br-Clm-7** was only measured three times.

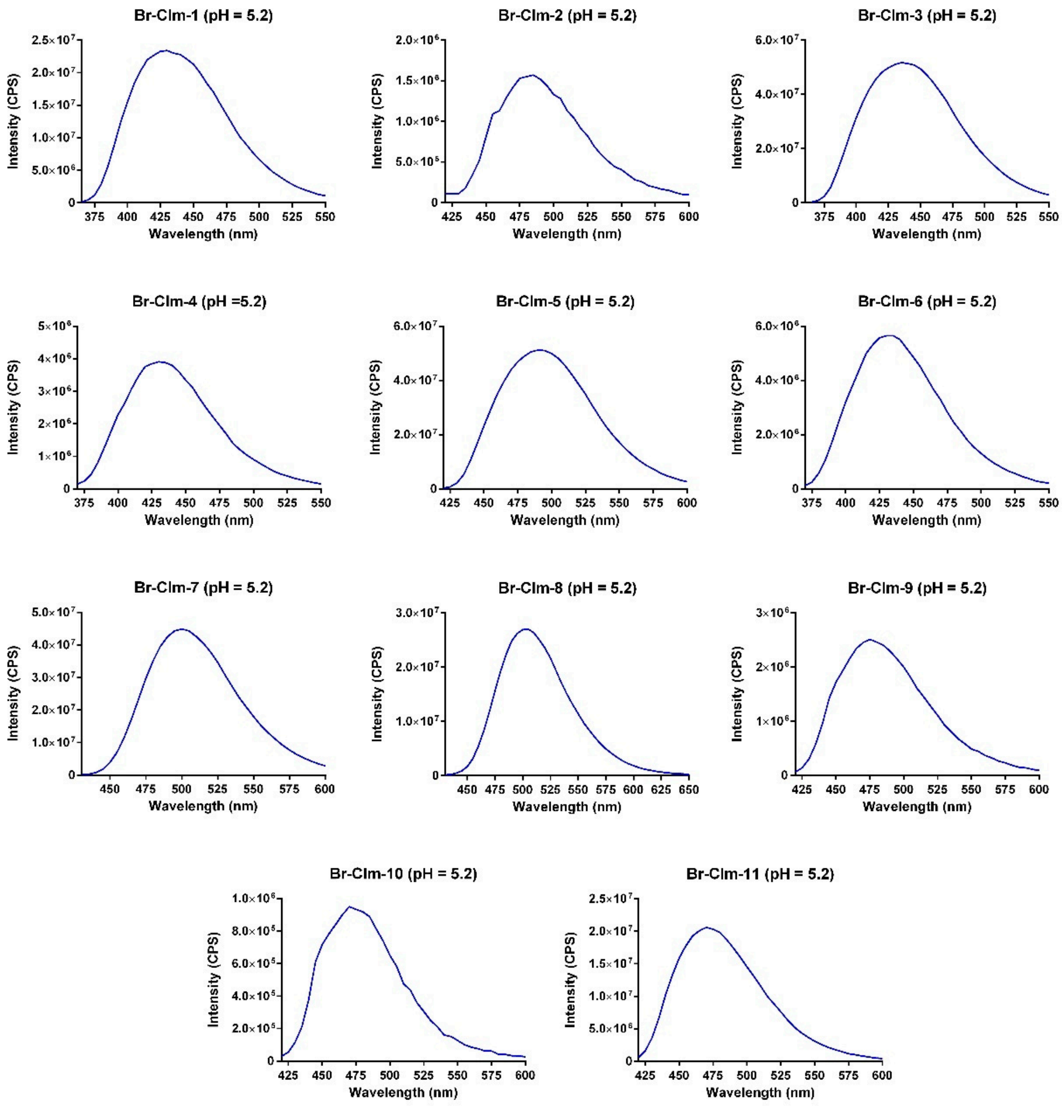

Figure S25. Emission spectra of the **Br-Clm** compounds in 30 μM aqueous solutions buffered to a pH of 5.2.

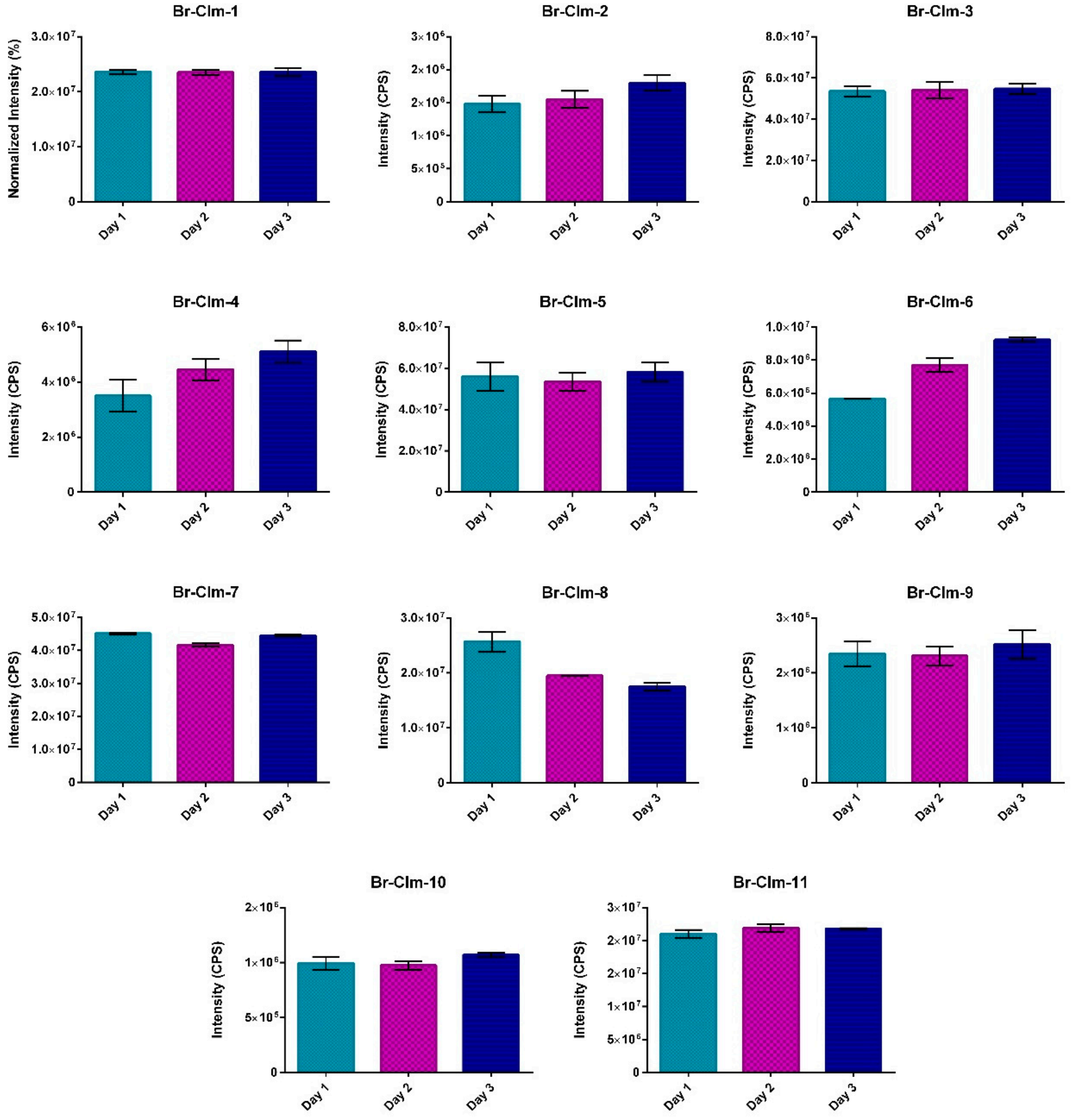

Figure S26. Maximum emission intensity of 30 μM aqueous solutions of the **Br-Clm** family buffered to a pH of 5.2, measured every 24 h.

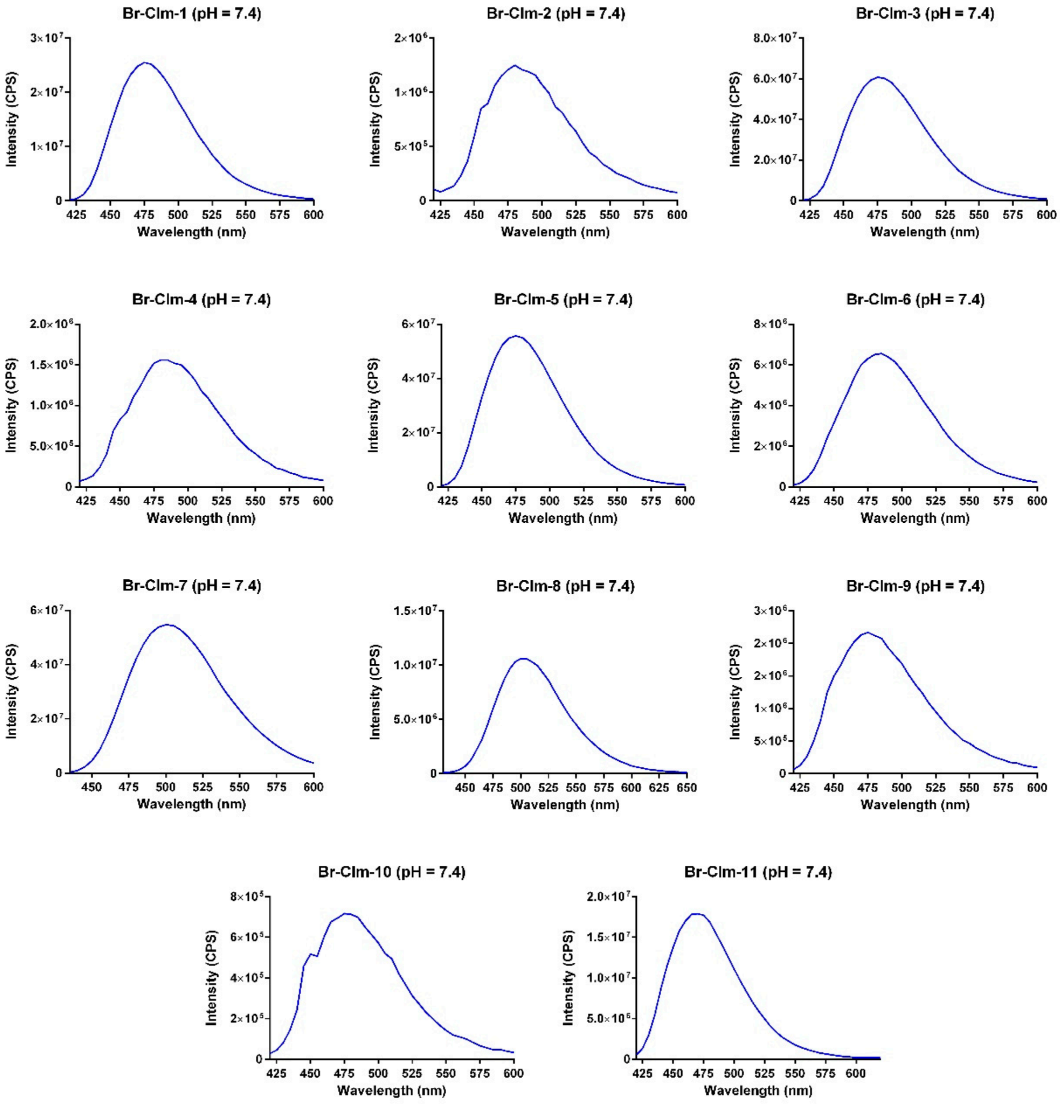

Figure S27. Emission spectra of the **Br-Clm** compounds in 30 μM aqueous solutions buffered to biological pH (7.4).

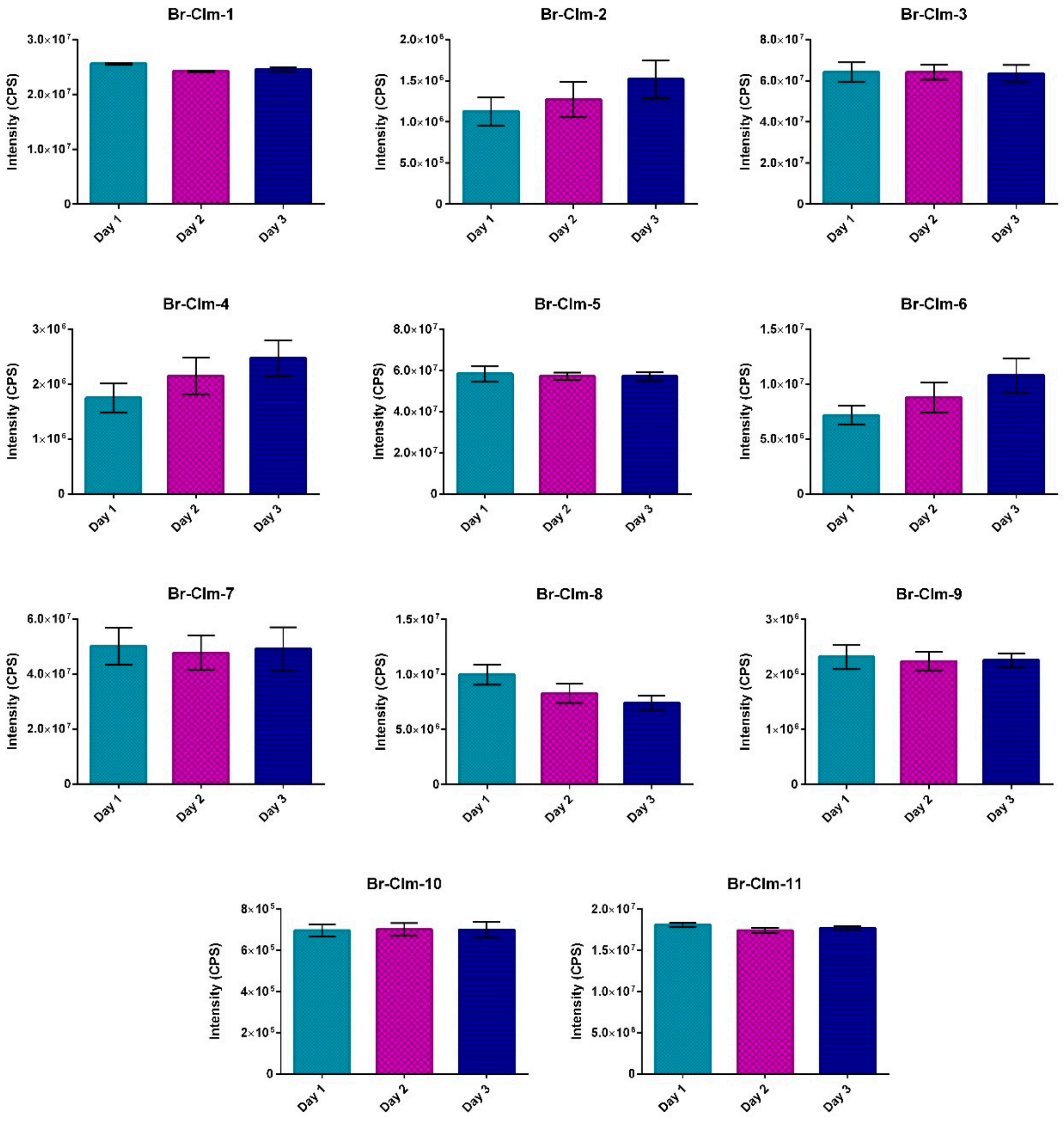

Figure S28. Maximum emission intensity of 30 μM aqueous solutions of the **Br-Clm** family buffered to a pH of 7.4, measured every 24 h.

4. Bibliography

1. Pinto da Silva, L.; Magalhães, C.M.; Núñez-Montenegro, A.; Ferreira, P.J.O.; Duarte, D.; Rodríguez-Borges, J.E.; Vale, N.; Esteves da Silva, J.C.G. Study of the Combination of Self-Activating Photodynamic Therapy and Chemotherapy for Cancer Treatment. *Biomolecules* **2019**, *9*, doi:10.3390/biom9080384.
2. Magalhães, C.M.; González-Berdullas, P.; Duarte, D.; Correia, A.S.; Rodríguez-Borges, J.E.; Vale, N.; Esteves da Silva, J.C.G.; Pinto da Silva, L. Target-Oriented Synthesis of Marine Coelenterazine Derivatives with Anticancer Activity by Applying the Heavy-Atom Effect. *Biomedicines* **2021**, *9*, doi:10.3390/biomedicines9091199.
